# FSCN1 Modulates Fatty Acid Metabolism and the Coordinated Activation of AKT/mTOR and p38 MAPK Pathways in Colorectal Cancer Cells

**DOI:** 10.32604/or.2026.084987

**Published:** 2026-09-14

**Authors:** Zhen Li, Xinya Yu, Boning Wu, Jialin Zhang, Xinyu Ju, Yajun Wang, Jieli Song, Qiao Liu, Peng Huang, Qi Ding, Yupeng Wu

**Affiliations:** 1 Bengbu Medical University Key Laboratory of Cancer Research and Clinical Laboratory Diagnosis, School of Laboratory Medicine, Bengbu Medical University, Bengbu, China; 2 Department of Blood Transfusion, The Affiliated Wuxi People’s Hospital of Nanjing Medical University, Wuxi People’s Hospital, Wuxi Medical Center, Nanjing Medical University, Wuxi, China; 3 Department of Biology, Hefei No.1 Middle School, Hefei, China; 4 State Key Laboratory of Oncology in South China, Collaborative Innovation Center for Cancer Medicine, Sun Yat-Sen University Cancer Center, Guangzhou, China; 5 Metabolic Innovation Center, Sun Yat-Sen University, Guangzhou, China; 6 School of Pharmacy, Bengbu Medical University, Bengbu, China; 7 Department of Biochemistry and Molecular Biology, School of Laboratory Medicine, Bengbu Medical University, Bengbu, China; 8 Anhui Provincial Key Laboratory of Tumor Evolution and Intelligent Diagnosis and Treatment, Bengbu Medical University, Bengbu, China

**Keywords:** Colorectal cancer, *FSCN1*, AKT/mTOR pathway, p38 MAPK pathway, metabolic regulation

## Abstract

**Background:** Fascin actin-bundling protein 1 (*FSCN1*) modulates the expression of key lipogenic enzymes fatty acid synthase (*FASN*) and stearoyl-CoA desaturase (*SCD1*) in colorectal cancer (CRC), but the underlying mechanisms remain elusive. **Methods:** Bioinformatics analyses were performed to evaluate FSCN1 expression and its prognostic value in CRC. Intracellular lipid levels following *FSCN1* knockdown were assessed by Nile Red/DAPI co-staining and triglyceride quantification, and further validated by Oil Red O staining of xenograft tumors. Expression levels of key metabolic enzymes were measured by qRT-PCR and Western blotting. RNA sequencing identified FSCN1-associated pathways, which were functionally investigated using pharmacological inhibitors. **Results:** FSCN1 was significantly upregulated in CRC (*p* < 0.001; AUC = 0.796) and was correlated with poorer overall survival (*p* = 0.018). FSCN1 depletion reduced intracellular lipid accumulation, accompanied by downregulation of lipogenic mediators—sterol regulatory element-binding transcription factor 1 (*SREBF1*; protein product: SREBP1), FASN, and SCD1—and upregulation of peroxisomal fatty acid oxidation (FAO)-related factors—peroxisome proliferator-activated receptor alpha (*PPARA*; protein product: PPARα) and acyl-CoA oxidase 1 (*ACOX1*). Mechanistically, FSCN1 was associated with activation of the protein kinase B/mammalian target of rapamycin (AKT/mTOR) and p38 mitogen-activated protein kinase (p38 MAPK) pathways; pharmacological inhibition with LY294002 or SB203580 phenocopied the lipid-lowering effects of *FSCN1* knockdown. **Conclusion:** Collectively, these findings link FSCN1 to the AKT/mTOR/SREBP1/(FASN/SCD1) lipogenic axis and the p38 MAPK/PPARα/ACOX1 peroxisomal FAO pathway, implicating FSCN1 in lipid metabolic regulation and CRC progression, while suggesting a putative functional regulatory axis and a promising candidate therapeutic target for CRC.

## 1 Introduction

Colorectal cancer (CRC) is the third most commonly diagnosed malignancy and the second leading cause of cancer-related mortality worldwide [1]. Distant metastasis is the primary contributor to CRC-related fatalities, and recent studies have indicated that the 5-year survival rate for patients with metastatic CRC is less than 20% [2,3], posing formidable challenges to patient survival and treatment. CRC progression from localized to metastatic disease involves complex molecular alterations, including metabolic reprogramming, which remains a major therapeutic challenge. Consequently, elucidating the molecular mechanisms driving CRC initiation and progression, and developing novel targeted therapies are essential for improving the cure and survival rates of CRC patients.

Metabolic reprogramming is a hallmark of cancer cell initiation and progression. The energy and intermediate metabolites generated through metabolic reprogramming contribute to cancer cell proliferation and invasion [4,5,6,7]. Lipid metabolic reprogramming is recognized as a key metabolic feature of cancer cells [8]. Dysregulation of key lipid metabolic enzymes disrupts cellular lipid homeostasis, subsequently affecting membrane architecture and signal transduction, and ultimately promoting metastatic competence [7,9,10,11]. For instance, in CRC, protein tyrosine phosphatase receptor type O (*PTPRO*) drives liver metastasis by coordinately regulating the lipid synthesis enzyme acetyl-CoA carboxylase 1 (*ACC1*) and lipid oxidation enzyme peroxisomal acyl-CoA oxidase 1 (*ACOX1*) to reprogram lipid metabolism [12]. In chronic lymphocytic leukemia, ACOX1-mediated peroxisomal FAO promotes metabolic reprogramming and resistance to caspase-dependent cell death, thereby promoting cell survival [13]. Therefore, maintaining lipid homeostasis is of paramount importance for human health [14], making lipid metabolic dysregulation a focal point in cancer research [15].

Fascin-1, encoded by the Fascin actin-bundling protein 1 (*FSCN1*) gene, is a 55 kDa cytoskeletal protein. By crosslinking with F-actin, it promotes the formation of membrane protrusions, including filopodia, lamellipodia, and microspikes, thereby enhancing tumor cell mobility, invasion, and metastasis [16]. Previous studies have shown that *FSCN1* downregulation inhibits tumor cell migration and invasion [17], whereas its upregulation promotes cancer cell motility [18]. In xenograft tumor models in nude mice, *FSCN1* overexpression enhances cancer cell metastatic potential [19], whereas its knockdown attenuates ovarian cancer metastasis [17]. Previous research demonstrated that Ly1 antibody reactive (LYAR) transcriptionally regulates *FSCN1* expression, subsequently increasing the expression of fatty acid synthase (*FASN*) and stearoyl-CoA desaturase 1 (*SCD1*), and promoting CRC cell invasion and metastasis [20]. However, the precise molecular mechanisms by which FSCN1 regulates lipid metabolism to facilitate CRC cell invasion and metastasis remain to be elucidated. This study aimed to elucidate the role of FSCN1 in promoting CRC progression via lipid metabolism regulating and the associated molecular mechanisms, which may provide potential strategies for the prevention and treatment of CRC.

## 2 Materials and Methods

### 2.1 Cell Lines and Cell Culture

The human colon cancer cell line HCT15 was purchased from the Typical Culture Preservation Commission Cell Bank, Chinese Academy of Sciences (Shanghai, China). The human colon cancer cell line RKO was gifted by Professor Mingrong Wang from the Cancer Hospital, Chinese Academy of Medical Sciences (Beijing, China). HCT15 and RKO cells were maintained on gelatinized 100 mm Cell Culture Dish (Cat. No. 704004; NEST, Wuxi, China) in RPMI-1640 (Cat. No. 31800022; Gibco, Waltham, MA, USA) and MEM Medium (Cat. No. 41500034; Gibco) supplemented with 10% fetal bovine serum (FBS; Cat. No. A5256701; Gibco) and 100 U/mL penicillin and 100 μg/mL streptomycin (Cat. No. 15140122; Gibco) at 37°C and 5% CO_2_. Both HCT15 and RKO cell lines were authenticated by short tandem repeat (STR) and confirmed to be free of cross-contamination and mycoplasma contamination. All experiments were performed using HCT15 and RKO cells at low passage numbers (no more than 15 passages post-thaw) to ensure their genetic and functional consistency.

### 2.2 Plasmids Construction and Viral Infection

To establish stable knockdown cells, short hairpin RNA (shRNA) oligonucleotides targeting *FSCN1* (*FSCN1*-shRNA1/2) and a non-targeting control (shNC) were designed based on the validated siRNA sequences from our previous study [20]. The oligonucleotides were synthesized by Tsingke Biotech (Beijing, China), annealed using a touchdown program in a PCR thermal cycler (Applied Biosystems, Foster City/Carlsbad, CA, USA) and then ligated into the *Xho*I (Cat. No. FD0694; Thermo Fisher Scientific, Waltham, MA, USA)/*Hpa*I (Cat. No. ER1031; Thermo Fisher Scientific) sites of the pLentiLox 3.7 lentiviral vector (7650 bp; Fitgene, Guangzhou, China), which carries a green fluorescent protein (GFP) reporter as the selection marker for positive cell sorting. Successful cloning was verified by DNA sequencing (Tsingke Biotech). The HCT15-*FSCN1*-KD1/KD2 stable cell lines were generated using these shRNA vectors in our prior study [20]; in the current study, the *FSCN1*-shRNA1 vector was used to generate the RKO-*FSCN1*-KD1 stable cell line. The shRNA sequences used were as follows: Human *FSCN1*-shNC-F: 5′-TTTCTCCGAACGTGTCACGTTTCAAGAGAACGTGACACGTTCGGAGAATTTTTTC-3′; Human *FSCN1*-shNC-R: 5′-TCGAGAAAAAATTCTCCGAACGTGTCACGTTCTCTTGAAACGTGACACGTTCGGAGAAA-3′; Human *FSCN1*-shRNA1-F: 5′-TGCGCCTACAACATCAAAGATTCAAGAGATCTTTGATGTTGTAGGCGCTTTTTTC-3′; Human *FSCN1*-shRNA1-R: 5′-TCGAGAAAAAAGCGCCTACAACATCAAAGATCTCTTGAATCTTTGATGTTGTAGGCGCA-3′; Human *FSCN1*-shRNA2-F: 5′-TGCCCATGATAGTAGCTTCATTCAAGAGATGAAGCTACTATCATGGGCTTTTTTC-3′; Human *FSCN1*-shRNA2-R: 5′-TCGAGAAAAAAGCCCATGATAGTAGCTTCATCTCTTGAATGAAGCTACTATCATGGGCA-3′.

To generate stable knockout cells, clustered regularly interspaced palindromic repeats (CRISPR)-Cas9 (CRISPR-associated 9) target sequences were designed using the Synthego online tool (http://www.synthego.com). The oligonucleotides corresponding to the target sites were phosphorylated, annealed, and cloned into the LentiCRISPRv2 green fluorescent protein (GFP) vector (13,131 bp; Tsingke Biotech), which uses GFP as the selection marker for stable cell isolation. The CRISPR-Cas9 target sequences were as follows:

Human *FSCN1*-sgRNA-F: 5′-CACCGGTTACCTGCTGTCTCCACCG-3′;

Human *FSCN1*-sgRNA-R: 5′-AAACCGGTGGAGACAGCAGGTAACC-3′.

Following co-transfection with vector components mixed at the ratios specified in the corresponding viral system manual, lentiviruses were packaged and produced in 293T cells. The viral supernatant was harvested, filtered through a sterile 0.45 μm membrane (Cat. No. HPWP01300; Millipore, Merck KGaA, Darmstadt, Germany), and used to infect the corresponding target cells. Successfully infected cells expressing GFP were sorted and purified via fluorescence-activated cell sorting (FACS) using a flow cytometer (Beckman, Brea, CA, USA) to establish homogeneous stable cell populations.

Of all stable cell lines used in this study, HCT15-*FSCN1*-KD1/KD2 were established and fully validated in our prior publication [20], while RKO-*FSCN1*-KD1 and HCT15-*FSCN1*-KO cell lines were newly generated in the current work using the protocols described above.

### 2.3 Quantitative Reverse Transcription Polymerase Chain Reaction (qRT-PCR)

Total RNA was isolated from the cells using the FastPure Cell/Tissue Total RNA Extraction Kit V2 (Cat. No. RC112-01; Vazyme, Nanjing, China). The RNA quality was verified by NanoDrop™ One spectrophotometry (Thermo Fisher Scientific), with OD_260_/OD_280_ and OD_260_/OD_230_ ratios of 1.9 and 2.1, respectively, meeting the optimal criteria for downstream applications. For complementary DNA (cDNA) synthesis, 1 μg of total RNA was reverse-transcribed using the HiScript II 1st Strand cDNA Synthesis Kit (+gDNA wiper; Cat. No. R212-01; Vazyme) according to the manufacturer’s protocol. qRT-PCR was performed on a Bio-Rad CFX96 system (Bio-Rad, Hercules, CA, USA) using 50 ng of cDNA template in a 20 μL reaction volume containing FastStart Universal SYBR Green Premix (Cat. No. 41106300; Roche, Basel, Switzerland). Gene-specific primers were designed using Primer Premier 6.0 software (PREMIER Biosoft International, Palo Alto, CA, USA) and synthesized by Tsingke Biotech. The primer sequences used in this study are listed in Table 1. β-actin (*ACTB*) served as the reference gene for normalization in CRC cells. Relative gene expression levels were calculated using the 2^−ΔΔCt^ method after normalizing to β-actin. Each sample was analyzed in biological triplicate, and all qPCR reactions were performed in technical triplicate.

**Table 1:** Sequences of primers used for qRT-PCR analysis.

Gene	Forward Primer	Reverse Primer
*ACTB*	5′-AGCACAGAGCCTCGCCTT-3′	5′-CTCGTCGCCCACATAGGAAT-3′
*FSCN1*	5′-CTGCTACTTTGACATCGAGTGG-3′	5′-GGGCGGTTGATGAGCTTCA-3′
*FASN*	5′-ACAGCGGGGAATGGGTACT-3′	5′-GACTGGTACAACGAGCGGAT-3′
*SCD1*	5′-CCCCACCTACAAGGATAAGGA-3′	5′-CACGAGCCCATTCATAGACAT-3′
*SREBF1*	5′-GCCCCTGTAACGACCACTG-3′	5′-CAGCGAGTCTGCCTTGATG-3′
*SREBF2*	5′-AACGGTCATTCACCCAGGTC-3′	5′-GGCTGAAGAATAGGAGTTGCC-3′
*PPARA*	5′-ATGGTGGACACGGAAAGCC-3′	5′-CGATGGATTGCGAAATCTCTTGG-3′
*PPARD*	5′-CAGGGCTGACTGCAAACGA-3′	5′-CTGCCACAATGTCTCGATGTC-3′
*ACOX1*	5′-ACTCGCAGCCAGCGTTATG-3′	5′-AGGGTCAGCGATGCCAAAC-3′
*CPT1A*	5′-TCCAGTTGGCTTATCGTGGTG-3′	5′-TCCAGAGTCCGATTGATTTTTGC-3′

### 2.4 Western Blotting

Cell extracts were prepared using RIPA buffer (Cat. No. P0013B; Beyotime, Shanghai, China) from CRC cell lines, and the cell lysates were quantified using an Enhanced BCA (Bicinchoninic Acid) Protein Assay Kit (Cat. No. P0010; Beyotime). Cell lysates (20–50 μg) were loaded and separated by 10–15% sodium dodecyl sulfate-polyacrylamide gel (Cat. No. PG111, PG112, PG113; Epizyme Biotech, Shanghai, China) and transferred onto nitrocellulose membranes (Cat. No. HATF00010; Millipore). After 1 h blocking with 5% No-fat milk blocking solution prepared with PBST (1× phosphate-buffered saline (PBS) with 0.1% Tween-20 (Cat. No. ST825; Beyotime)), the membranes were incubated with the primary antibodies overnight at 4°C. Detailed information on all primary antibodies, including their dilution ratios and catalog numbers, is provided in Table 2. With β-actin as the internal control, the membranes were subsequently incubated with a secondary antibody (Beyotime) for 1 h at room temperature. The specific bands were visualized using a chemiluminescence reagent (Cat. No. BL523B; Biosharp, Beijing, China) in a BeyoImager™600 Chemiluminescence Imaging System (Beyotime), and band intensities were quantified using ImageJ software (v1.8.0; National Institutes of Health, Bethesda, MD, USA). The quantification was performed in a blinded manner: using ImageJ, identical parameters were applied throughout the densitometric analysis, and no distinction was made between control and experimental groups during the measurement phase. Only after the gray values of all bands had been measured were the data assigned to their respective groups, thereby eliminating subjective bias.

**Table 2:** Primary antibodies used for Western blotting.

Antibody	Company (Cat. No.)	Working Dilutions
FSCN1	Proteintech (66321-1-Ig)	WB: 1:2000
FASN	Proteintech (10624-2-AP)	WB: 1:20,000
SCD1	Proteintech (28678-1-AP)	WB: 1:8000
CPT1A	Proteintech (15184-1-AP)	WB: 1:1000
ACOX1	Proteintech (10957-1-AP)	WB: 1:4000
AKT	Proteintech (10176-2-AP)	WB: 1:1000
Phospho-AKT (Ser473)	Proteintech (66444-1-Ig)	WB: 1:5000
p38 MAPK	Proteintech (14064-1-AP)	WB: 1:1000
Phospho-p38 MAPK (Thr180/Tyr182)	Proteintech (28796-1-AP)	WB: 1:2000
ERK1/2	Proteintech (11257-1-AP)	WB: 1:8000
Phospho-ERK1/2 (Thr202/Tyr204)	Proteintech (28733-1-AP)	WB: 1:3000
mTOR	Proteintech (66888-1-Ig)	WB: 1:20,000
Phospho-mTOR (Ser2448)	Proteintech (67778-1-Ig)	WB: 1:10,000
SREBP1	Proteintech (14088-1-AP)	WB: 1:2000
PPARα	Proteintech (66826-1-Ig)	WB: 1:3000
β-actin	Proteintech (20536-1-AP)	WB: 1:5000

### 2.5 Nile Red Staining

Cells were fixed with 4% paraformaldehyde (PFA) (Cat. No. BL539A; Biosharp) and stained with Nile Red (Cat. No. N8440; Solarbio, Beijing, China) according to the protocol described below. Briefly, a Nile Red stock solution (1 mg/mL) was prepared by dissolving the dye in dimethyl sulfoxide (DMSO; Cat. No. D8371; Solarbio). For cellular staining, the stock solution was diluted to a final working concentration of 10 μM, and 1 mL of this working solution was added to each well, followed by incubation for 30 min in the dark. After staining, the cells were rinsed thoroughly with 1× PBS (Cat. No. BL302A; Biosharp). Subsequently, the cells were counterstained with a working solution of the DNA-binding fluorochrome 4′,6-diamidino-2-phenylindole (DAPI; Cat. No. BL105A; Biosharp). Fluorescence images were captured using an inverted fluorescence microscope (Thermo Fisher Scientific) at a magnification of 200×. A blinded analysis was performed for Nile Red staining to eliminate observer bias. Subsequently, the integrated optical density (IOD) of the positively stained regions was quantified using ImageJ software. Valid quantification ranges for optical density and area were defined by threshold calibration against negative controls. To ensure specificity, only fluorescent regions surpassing the background and displaying characteristic lipid droplet morphology were included, thereby minimizing false positives from nonspecific staining. To account for cell density differences, the IOD values were normalized to the number of cells per field of view, yielding the IOD per cell. The relative red fluorescence intensity, which reflects the intracellular lipid droplet content, was calculated from these measurements. Data were based on three independent biological replicates, and statistical analysis was conducted using GraphPad Prism software (v9.0; GraphPad Inc., Boston, MA, USA).

### 2.6 Oil Red O (ORO) Staining

Preparation of ORO Stock Solution: ORO powder (Cat. No. O0625; Sigma-Aldrich, St. Louis, MO, USA) was dissolved in a chloroform/ethanol mixture (1:1, v/v) to a concentration of 1 mg/mL, followed by incubation in the dark for 15 min. The stock solution was then diluted with double-distilled water (ddH_2_O) to prepare an ORO working solution at a final concentration of 60% (v/v), which was filtered prior to use. Mouse xenograft tissues were derived from the subcutaneous implantation of HCT15-*FSCN1*-NC (negative control, *n* = 6) and HCT15-*FSCN1*-KD1 (knockdown, *n* = 6) cells. Tissues were fixed in 4% PFA, cut into appropriate-sized blocks, and dehydrated sequentially in 20% and 30% sucrose solutions (1 day per gradient). Dehydrated tissues were embedded in optimal cutting temperature (OCT) compound, snap-frozen, and sectioned into 8-μm-thick slices. For staining, the slices were first briefly immersed in 60% isopropanol, then incubated in the ORO working solution for 15 min at room temperature in the dark. After staining, the slices were rinsed twice with 60% isopropanol and twice with ddH_2_O for washing. Cell nuclei were counterstained with hematoxylin (Cat. No. 51275; Sigma-Aldrich) for 10 s, followed by thorough rinsing with ddH_2_O and air-drying. Finally, the stained sections were imaged using an inverted microscope (Thermo Fisher Scientific). Consistent with the blind statistical analysis applied to the Nile Red staining results described above, a blind analysis was also performed for the ORO staining assay data.

### 2.7 Triglyceride Quantification

Cell samples were thoroughly washed with 1× PBS to eliminate residual culture medium and cell surface contaminants, and the resultant cells were then lysed to achieve complete release of intracellular triglycerides into the lysate. The triglyceride content of the samples was quantified by measuring the absorbance using a triglyceride assay kit (Cat. No. A110-1-1; Nanjing Jiancheng, Nanjing, China). The protein concentration of the cell lysate was determined using the BCA assay. To enable reliable comparison across different samples, the measured triglyceride values were normalized to the protein concentration to obtain absolute levels (mmol/g protein). Subsequently, these absolute values were normalized to the mean value of the corresponding control group (set as 1.0) to determine relative fold changes, which are presented in the figures as ‘relative triglyceride content’. All experiments were conducted in triplicate to ensure data reproducibility. Statistical analyses were performed using GraphPad Prism software.

### 2.8 Inhibitors and Vehicle Control

LY294002 (Cat. No. S1105; Selleck Chemicals, Houston, TX, USA): One milligram of the inhibitor was dissolved in 0.0651 mL DMSO to prepare a stock solution. A final working concentration of 20 μM was used for cell treatment, as previously reported [21]. Cells were treated with LY294002 at this concentration and incubated for 48 h in a humidified incubator at 37°C with 5% CO_2_.

SB203580 (Cat. No. S1076; Selleck Chemicals): One milligram of the inhibitor was dissolved in 0.0530 mL DMSO to prepare the stock solution. According to published data [22], a final working concentration of 20 μM was used for cell treatment. The cells were incubated with SB203580 at the aforementioned concentration for 48 h under the same conditions (37°C, 5% CO_2_).

The cell culture medium of the vehicle control group was supplemented with DMSO at the same final concentration as that used in the inhibitor-treated experimental groups. The cells were incubated under identical conditions for 48 h without the addition of inhibitors.

### 2.9 Public Databases

The mRNA expression profiles and clinical data of CRC tissues were downloaded from The Cancer Genome Atlas Program (TCGA, https://portal.gdc.cancer.gov/; dbGaP accession: phs000178; Supplementary Material S1: TCGA-COADREAD) database. Additionally, mRNA expression profiles of normal colorectal mucosal tissues were retrieved from the Genotype-Tissue Expression (GTEx, https://gtexportal.org/home/; dbGaP accession: phs000424) database. Level-3 HTSeq FPKM format data were first normalized to transcripts per million (TPM; Supplementary Material S2: TPM_normalize_COADREAD), and then subjected to Log_2_ (TPM + 1) transformation for subsequent pan-cancer analysis and data visualization. For all survival endpoint analyses, cases with incomplete overall survival follow-up information were excluded from the final analyzable cohort, in accordance with standard filtering conventions for cancer prognostic research.

### 2.10 GSEA Pathway Enrichment

Gene set enrichment analysis (GSEA) was performed using the “clusterProfiler” R software package (v4.3.1). The input consisted of a pre-ranked list of all expressed genes, ordered by their signed log_2_ fold change values calculated from the differential expression analysis of public transcriptomic datasets. The gene sets used for the enrichment analysis were sourced from the Kyoto Encyclopedia of Genes and Genomes (KEGG), WikiPathways, and Reactome databases. Functional categories or pathways were deemed significantly enriched when the following criteria were satisfied: adjusted *p*-value < 0.05 and false discovery rate (FDR) < 0.25.

### 2.11 Animal Experiments

All animal experiments were designed, performed, and reported in compliance with the ARRIVE 2.0 Essential 10 guidelines (https://arriveguidelines.org/resources/author-checklists). All experimental protocols were reviewed and approved by the Experimental Animal Management and Ethics Committee of Bengbu Medical University (Approval No. 656 [2023]).

Six-week-old female BALB/c nude (nu/nu) mice were purchased from Beijing Weitonglihua Experimental Animal Technology Co., Ltd. (Beijing, China) and housed under specific pathogen-free (SPF) conditions at the Experimental Animal Center of Bengbu Medical University, with a 12 h light/dark cycle, controlled ambient temperature (22 ± 2°C), relative humidity (50 ± 10%), and ad libitum access to sterilized standard chow and drinking water.

For the subcutaneous tumor xenograft assay, mice were randomly assigned to two experimental groups (*n* = 6 mice per group) and subcutaneously implanted with 1 × 10^6^ stable HCT15 cell clones (*FSCN1*-NC or *FSCN1*-KD1) in the right dorsal flank. Tumor length and width were measured every 3 days using digital calipers, and tumor volume was calculated according to the formula: V = (length × width^2^)/2. Mice were monitored daily for general health and behavioral status; pre-established humane endpoints approved by the ethics committee included >20% body weight loss, maximum tumor diameter exceeding 15 mm, or signs of severe pain and distress (e.g., persistent hunched posture, impaired mobility, self-mutilation).

The mice were monitored for 30 days. At the end of the experiment, all mice were first deeply anesthetized via inhalation of 3–4% isoflurane, a short-acting volatile anesthetic with no documented significant effect on core tumor lipid metabolism endpoints such as triglyceride content and lipid droplet profiles, and then humanely euthanized via cervical dislocation in strict accordance with the ethics committee-approved animal welfare protocol. Subsequently, the xenograft tumors were surgically excised. Each tumor specimen was immediately cryopreserved and processed into frozen sections to facilitate the quantitative analysis of intracellular lipid content.

### 2.12 Statistical Analysis

All statistical analyses were conducted using R software (v4.3.1; R Foundation for Statistical Computing, Vienna, Austria) and GraphPad Prism (v9.0; GraphPad Inc., Boston, MA, USA). The R base package stats was used for basic hypothesis testing, and the multcomp package (v1.4-25) was employed for post hoc multiple comparisons. Each experiment was performed with three independent biological replicates (*n* = 3). Comparisons between two independent groups were performed using a two-tailed Student’s *t*-test, whereas comparisons among three or more groups were analyzed using one-way analysis of variance (ANOVA) followed by Tukey’s honestly significant difference (HSD) test for post hoc pairwise comparisons. When multiple independent *t*-tests were applied across different experimental comparisons, the Benjamini–Hochberg false discovery rate (FDR) correction was applied to control for Type I error. A *p*-value < 0.05 was considered statistically significant. Statistical significance was denoted as *p < 0.05, **p < 0.01, ***p < 0.001, and ****p < 0.0001.

## 3 Results

### 3.1 Overexpression of FSCN1 in CRC Tissues and Its Clinical Significance

**Figure 1:**
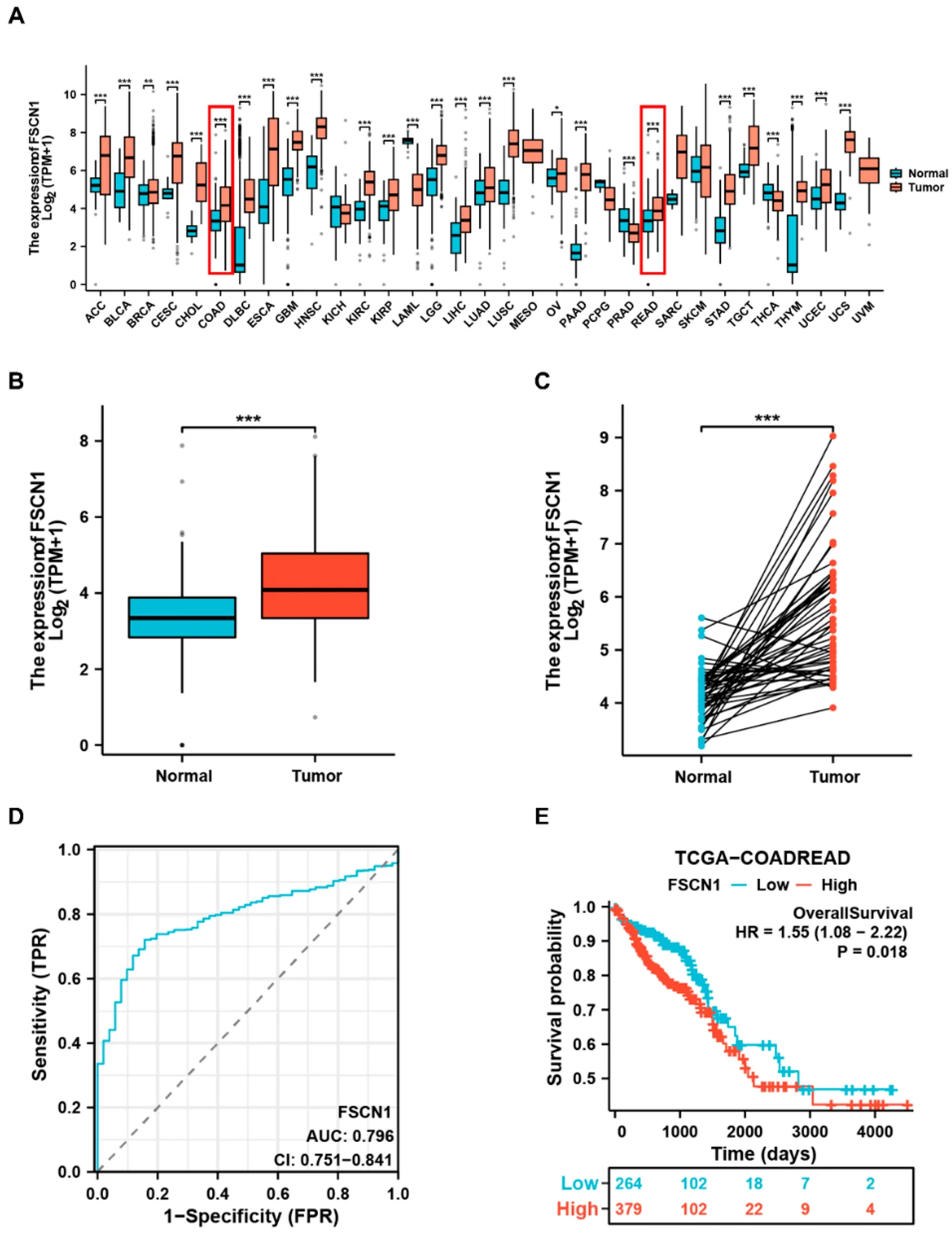
High FSCN1 expression in colorectal cancer (CRC) and its association with poor prognosis. (**A**) Pan-cancer analysis comparing FSCN1 expression in TCGA tumor samples versus GTEx normal tissues, revealing tumor-specific overexpression across multiple cancer types. The red boxes specifically highlight colon adenocarcinoma (COAD) and rectum adenocarcinoma (READ), showing significantly elevated FSCN1 expression in colorectal cancer tissues compared to normal counterparts. (**B**) Elevated FSCN1 transcripts in CRC tumors (TCGA-COADREAD) relative to GTEx-matched normal colorectal mucosa (Normal = 51, Tumor = 644). (**C**) Paired tumor/normal tissue analysis confirming FSCN1 upregulation in CRC patients (Normal = 50, Tumor = 50). (**D**) ROC curve demonstrating the diagnostic potential of FSCN1 (AUC = 0.796, 95% CI: 0.751–0.841) for differentiating CRC from normal mucosa. (**E**) Kaplan-Meier survival curves showing reduced overall survival in patients with high FSCN1 expression. Note: One tumor sample was excluded from the survival analysis due to unavailable clinical follow-up data, resulting in n = 643 for this panel. *p < 0.05; **p < 0.01; ***p < 0.001. Abbreviations: FSCN1, fascin actin-bundling protein 1; TCGA, The Cancer Genome Atlas; COADREAD, colon adenocarcinoma and rectal adenocarcinoma; TPM, transcripts per million; AUC, area under the curve; CI, confidence interval; HR, hazard ratio; FPR, false positive rate; TPR, true positive rate.

To investigate *FSCN1* expression patterns in CRC, comprehensive bioinformatics analyses were performed, followed by experimental validation. Pan-cancer analysis demonstrated ubiquitous FSCN1 upregulation across multiple malignancies, with particularly pronounced expression in colorectal, cervical, esophageal, and lung squamous cell carcinomas (Fig. 1A). Comparative analysis revealed striking differences in FSCN1 expression between malignant and normal tissue. CRC tissues exhibited significantly elevated FSCN1 levels compared to normal colorectal tissues (*p* < 0.001; Fig. 1B). Paired analysis showed consistent FSCN1 overexpression in tumor tissues compared to adjacent normal mucosa from the same patients (*p* < 0.001; Fig. 1C). The diagnostic potential of FSCN1 was evidenced by the area under the curve (AUC) of the receiver operating characteristic curve (ROC) (AUC = 0.796, 95% CI: 0.751–0.841), indicating a robust discriminative power for distinguishing CRC from normal mucosa (Fig. 1D). Notably, Kaplan-Meier survival analysis revealed significantly worse overall survival in patients with high FSCN1 expression (*p* = 0.018; Fig. 1E). Collectively, these findings indicate that *FSCN1* is consistently overexpressed in CRC tissues and that high *FSCN1* expression may be significantly associated with a poor prognosis.

### 3.2 FSCN1 Depletion Reduces Lipid Accumulation in CRC Cells

**Figure 2:**
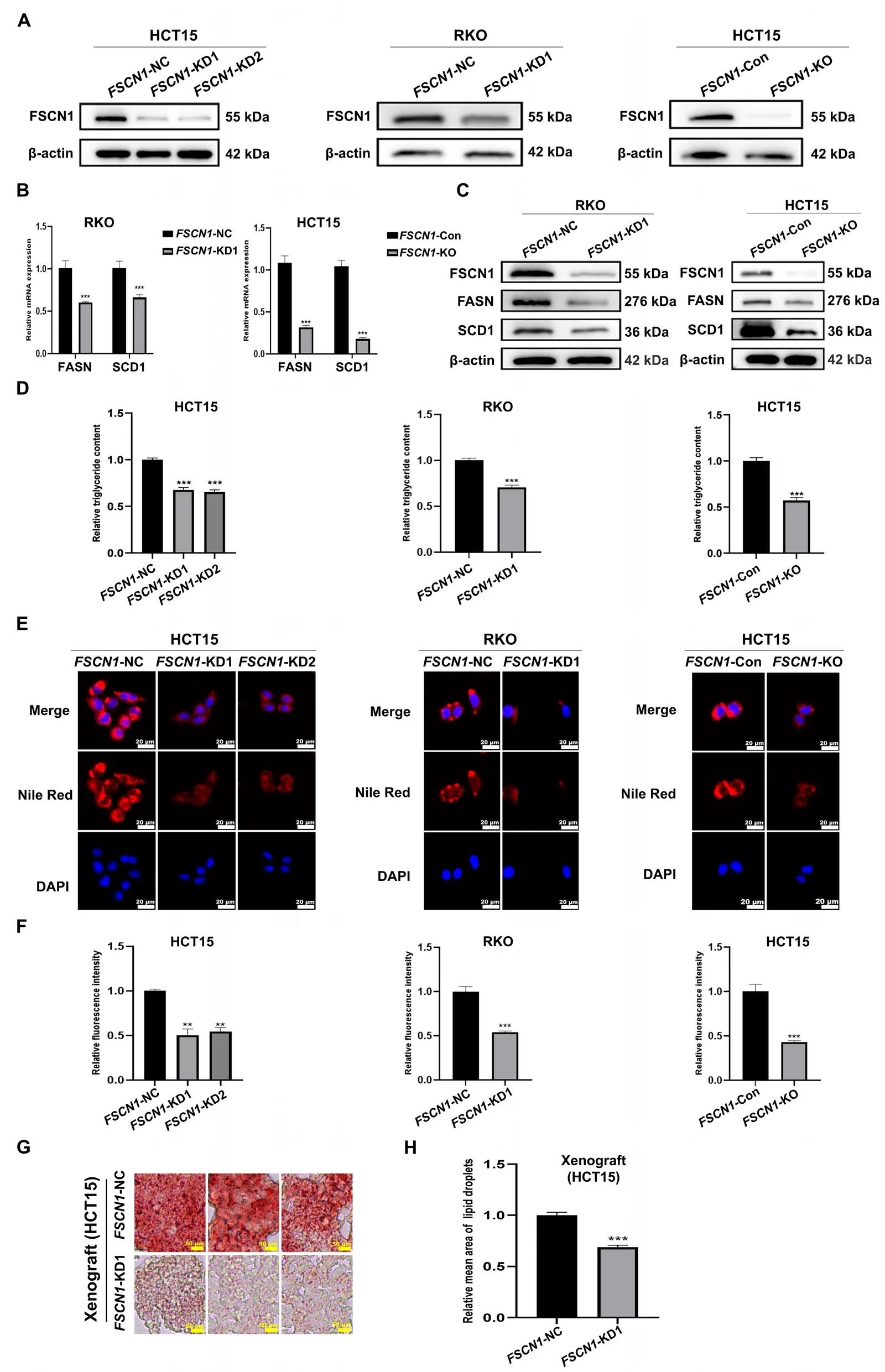
*FSCN1* knockdown significantly reduces lipid accumulation in colorectal cancer (CRC) cells. (**A**) Western blot analysis of FSCN1 protein levels in stable *FSCN1*-KD/KO cell lines and corresponding controls, with corresponding densitometric quantification in Supplementary Fig. S2. (**B**) qRT-PCR analysis of FASN and SCD1 mRNA levels normalized to β-actin in stable *FSCN1*-KD/KO cell lines and corresponding controls. (**C**) Western blot analysis of FASN and SCD1 protein levels in stable *FSCN1*-KD/KO cell lines and corresponding controls, with corresponding densitometric quantification in Supplementary Fig. S3. (**D**) Quantitative analysis of relative triglyceride content in stable *FSCN1*-KD/KO cell lines and corresponding controls. (**E**) Representative fluorescence images of cells co-stained with Nile Red (red fluorescence; a hydrophobic dye that specifically binds to intracellular lipid droplets and neutral lipids) and DAPI (blue fluorescence; a nuclear counterstain). Blinded quantitative analysis was performed for Nile Red staining. The staining shows the overall trend of neutral lipid distribution, with lipid droplet quantification independently validated by triglyceride assays. Scale bar: 20 μm. (**F**) Relative Nile red fluorescence intensity of cells in panel (**E**), normalized to DAPI staining intensity. (**G**) Representative ORO staining images of frozen HCT15 xenograft tumor sections from three independent mice per group (nuclei counterstained with hematoxylin). Blinded quantitative analysis was conducted for ORO staining. Scale bar: 50 μm. (**H**) Quantification of the average relative area of ORO-stained intracellular lipid droplets, corresponding to the images in panel (**G**). β-actin served as the loading control. All quantitative data are presented as mean ± standard deviation (SD) from three independent biological replicates (*n* = 3). **p < 0.01; ***p < 0.001. Abbreviations: FSCN1, fascin actin-bundling protein 1; FASN, fatty acid synthase; SCD1, stearoyl-CoA desaturase 1; NC, negative control; Con, control; KD, knockdown; KO, knockout; DAPI, 4′,6-diamidino-2-phenylindole.

Building upon our previous findings—in which stable *FSCN1*-knockdown HCT15 cell lines (HCT15-*FSCN1*-KD1/KD2) were established and fully validated, and FSCN1 was shown to modulate key lipogenic enzymes FASN and SCD1 in HCT15 cells [20]—we newly generated stable *FSCN1*-knockdown RKO cells (RKO-*FSCN1*-KD1, using the same target sequence as HCT15-*FSCN1*-KD1) and *FSCN1*-knockout HCT15 cells (HCT15-*FSCN1*-KO) in the present study to further investigate the regulatory role of FSCN1 in lipid metabolism. Successful modulation of *FSCN1* expression in these newly established cell lines was confirmed at both the transcriptional and protein levels (Fig. 2A and Supplementary Figs. S1 and S2). Consistent with previous findings, knockdown or knockout of *FSCN1* markedly decreased FASN and SCD1 expression in both HCT15 and RKO CRC cell lines (Fig. 2B,C and Supplementary Fig. S3). However, whether changes in FSCN1 expression affect lipid levels in CRC cells remains unknown. To address this, quantitative triglyceride assays were performed. The results demonstrated a significantly decreased triglyceride content in *FSCN1*-knockdown and *FSCN1*-knockout cells (Fig. 2D). Furthermore, the lipophilic dye Nile Red was applied to stain and analyze cellular lipids. Compared with control cells, the Nile Red fluorescence signal (red) was markedly weaker in *FSCN1*-knockdown and *FSCN1*-knockout cells, indicating that *FSCN1* suppression significantly diminished lipid accumulation (Fig. 2E,F). These *in vitro* findings were consistent with results from xenograft models generated using the validated HCT15-*FSCN1*-KD1 cell line, in which FSCN1 exhibits a significant tumor-promoting effect [20]. Oil Red O (ORO) staining revealed significantly lower ORO staining area in xenograft tumors derived from *FSCN1*-knockdown HCT15 cells compared with controls (Fig. 2G,H). Collectively, these results show that FSCN1 expression correlates with upregulated FASN and SCD1 levels and increased lipid accumulation in CRC cells, suggesting a potential role for FSCN1 in lipid anabolic regulation.

### 3.3 FSCN1 Expression Correlates with SREBF1 Levels and Lipid Synthesis in CRC Cells

**Figure 3:**
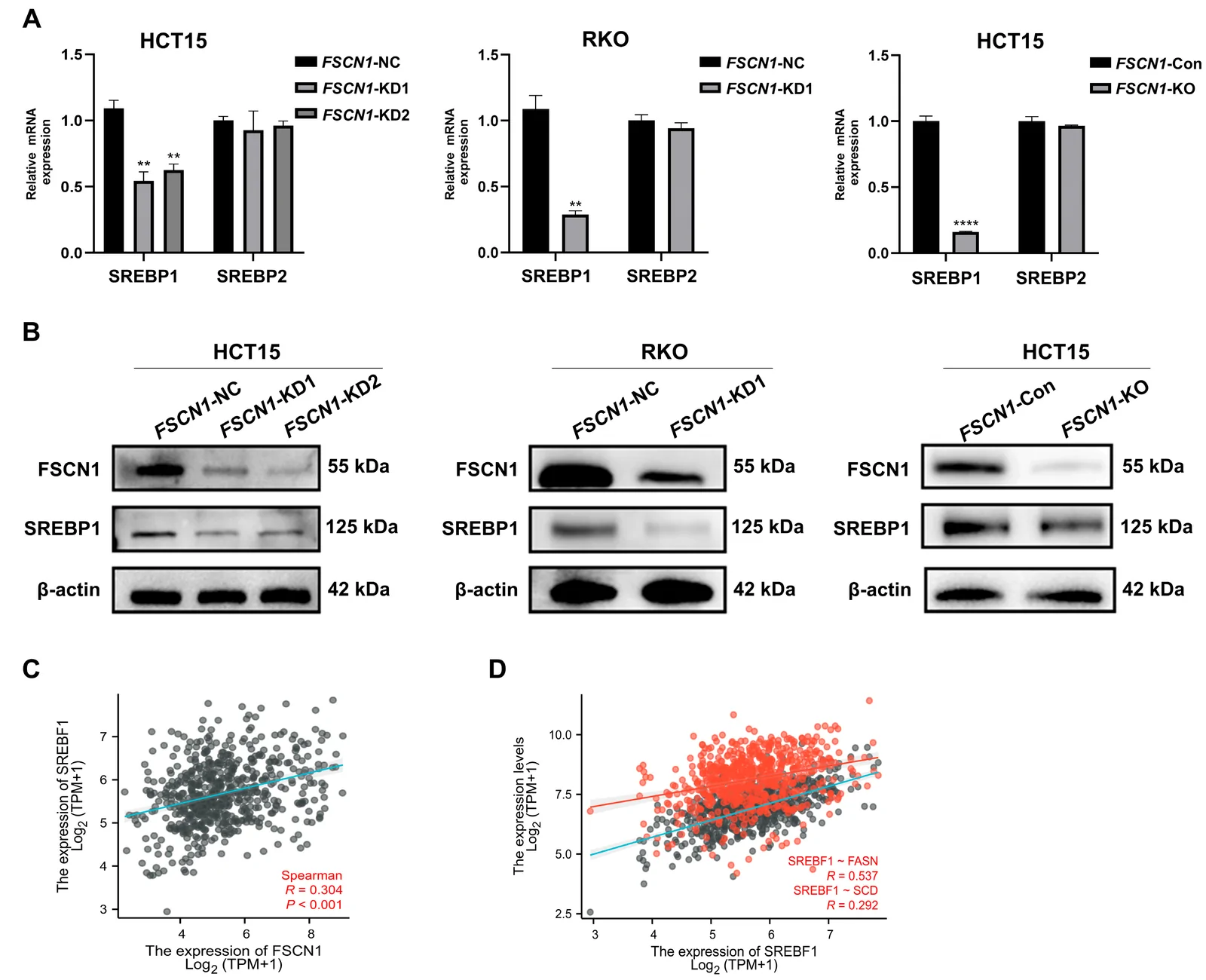
FSCN1 expression correlates with SREBP1 levels and lipid synthesis in colorectal cancer (CRC) cells. (**A**) qRT-PCR analysis of SREBP1 and SREBP2 mRNA levels normalized to β-actin in stable *FSCN1*-KD/KO cell lines and corresponding controls. (**B**) Western blot analysis of SREBP1 protein level in stable *FSCN1*-KD/KO cell lines and corresponding controls, with corresponding densitometric quantification in Supplementary Fig. S4. (**C**) Clinicogenomic correlation analysis (TCGA-COADREAD cohort) showing a positive association between FSCN1 and SREBP1 (encoded by *SREBF1*) expression. (**D**) Validation of transcriptional correlation between SREBP1 (encoded by *SREBF1*) and its downstream lipogenic targets FASN and SCD1 (also known as SCD) in an independent CRC cohort. β-actin served as the loading control. Data are shown as the mean ± standard deviation (SD) from three independent biological replicates (*n* = 3). **p < 0.01; ****p < 0.0001. Abbreviations: FSCN1, fascin actin-bundling protein 1; SREBP1, sterol regulatory element-binding protein 1; SREBP2, sterol regulatory element-binding protein 2; NC, negative control; KD, knockdown; KO, knockout; Con, control; β-actin, beta-actin; TPM, transcripts per million.

Previous studies have demonstrated that sterol regulatory element-binding transcription factors (SREBFs) function as trans-activators of key enzymes in lipid biosynthesis [23]. To explore whether SREBFs mediate FSCN1-induced upregulation of FASN and SCD1 in CRC cells, mRNA expression of the two major SREBF family members, SREBP1 (*SREBF1*) and SREBP2 (*SREBF2*), was initially analyzed, using qRT-PCR. The results showed that SREBP1 mRNA expression was significantly downregulated in *FSCN1*-knockdown and *FSCN1*-knockout cells, whereas SREBP2 mRNA expression remained unchanged (Fig. 3A). Western blot analysis further confirmed that SREBP1 protein expression was substantially decreased following *FSCN1* suppression in CRC cells (Fig. 3B and Supplementary Fig. S4). Additionally, bioinformatics analysis revealed a positive correlation between FSCN1 and SREBP1 (*SREBF1*) expression (Fig. 3C), as well as between SREBP1 (*SREBF1*) expression and FASN and SCD1 expression (Fig. 3D). In summary, our results demonstrate that FSCN1 upregulates SREBP1 expression, which is known to transcriptionally activate FASN and SCD1 expression. This regulatory cascade may contribute to promoting lipid synthesis and be associated with altered intracellular lipid accumulation in CRC cells.

### 3.4 FSCN1 Expression Is Inversely Associated with Peroxisomal FAO in CRC Cells

**Figure 4:**
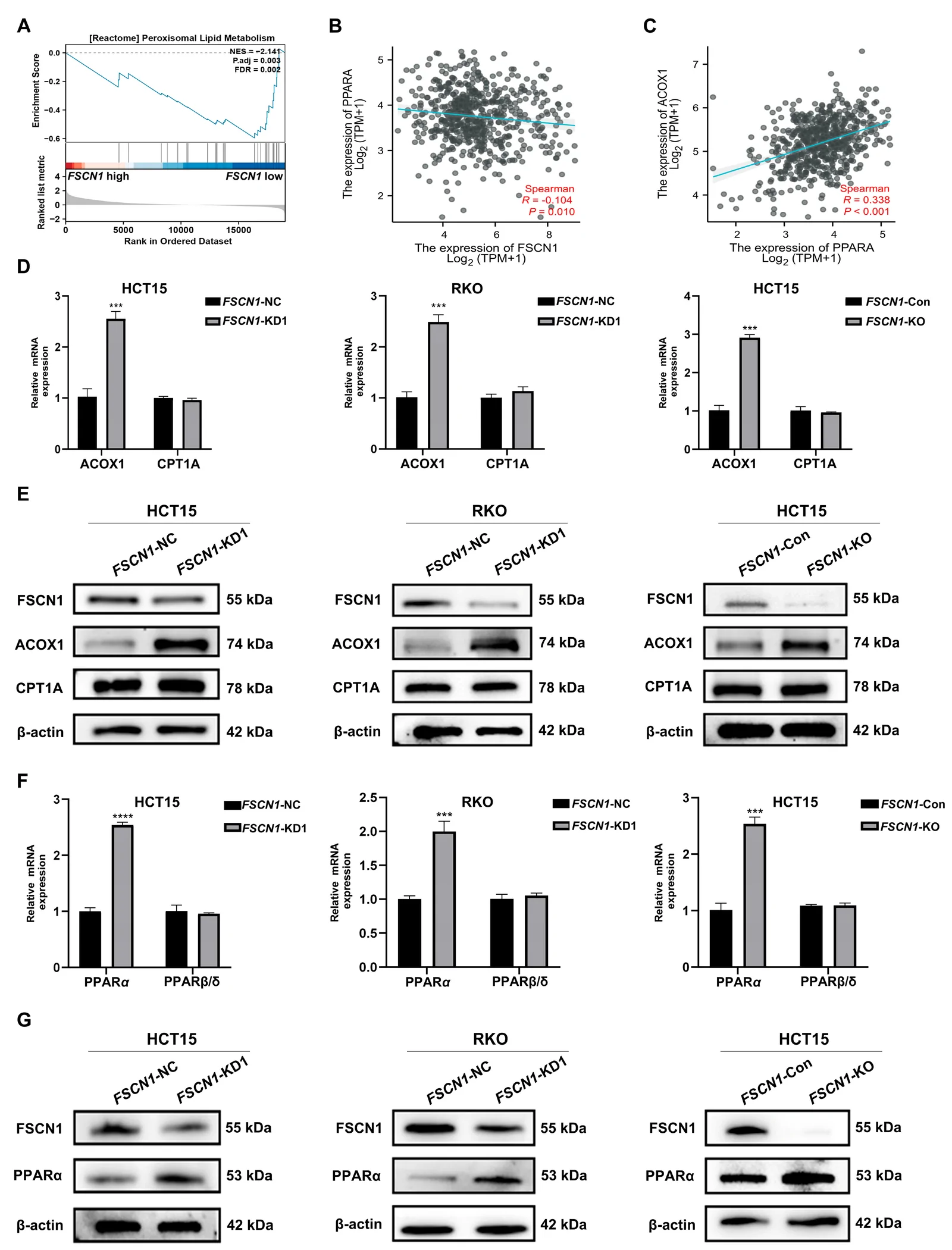
FSCN1 expression is inversely associated with peroxisomal FAO pathway in colorectal cancer (CRC) cells. (**A**) GSEA (Reactome database) of the TCGA-COADREAD cohort reveals significant suppression of the peroxisomal lipid pathway, indicating reduced FAO activity, in FSCN1-high tumors (FDR < 0.25, *p* < 0.05). (**B**) Clinicogenomic correlation analysis (TCGA-COADREAD cohort) revealing a negative association between FSCN1 and PPARα (encoded by *PPARA*) expression. (**C**) Validation of transcriptional correlation between PPARα (encoded by *PPARA*) and ACOX1 in an independent CRC cohort. (**D**) qRT-PCR analysis of CPT1A and ACOX1 (two key FAO enzymes) mRNA levels normalized to β-actin in stable *FSCN1*-KD/KO cell lines and corresponding controls. (**E**) Western blot analysis of CPT1A and ACOX1 protein expression in stable *FSCN1*-KD/KO cell lines and corresponding controls, with corresponding densitometric quantification in Supplementary Fig. S5. (**F**) qRT-PCR analysis of PPARα and PPARβ/δ (key FAO regulators) mRNA levels normalized to β-actin in stable *FSCN1*-KD/KO cell lines and corresponding controls. (**G**) Western blot analysis of PPARα protein expression in stable *FSCN1*-KD/KO cell lines and corresponding controls, with corresponding densitometric quantification in Supplementary Fig. S6. β-actin served as the loading control. Data are shown as the mean ± standard deviation (SD) from three independent biological replicates (*n* = 3). ***p < 0.001; ****p < 0.0001. Abbreviations: GSEA, Gene Set Enrichment Analysis; NES, normalized enrichment score; FDR, false discovery rate; FSCN1, Fascin actin-bundling protein 1; PPARα, peroxisome proliferator-activated receptor alpha; PPARβ/δ, peroxisome proliferator-activated receptor beta/delta; ACOX1, acyl-CoA oxidase 1; CPT1A, carnitine palmitoyltransferase 1A; NC, negative control; KD, knockdown; KO, knockout; Con, control; *β*-actin, beta-actin.

To explore the potential role of FSCN1 in FAO within CRC cells, Gene Set Enrichment Analysis (GSEA) was conducted using the TCGA-COADREAD dataset. The results showed that high FSCN1 expression was negatively correlated with FAO rates in CRC tissues (Fig. 4A). Subsequently, qRT-PCR and Western blot analyses were conducted to examine key FAO enzymes. The findings revealed that inhibiting *FSCN1* expression significantly increased *ACOX1* expression, whereas carnitine palmitoyltransferase 1 (*CPT1A*) expression remained unchanged (Fig. 4D,E and Supplementary Fig. S5). Previous studies have established that peroxisome proliferator-activated receptors (PPARs) are core transcriptional regulators of FAO and play critical roles in lipid metabolism [24]. To explore whether PPARs are involved in the FSCN1-associated alterations in ACOX1 expression in CRC cells, mRNA levels of two major PPAR family members, PPARα and PPARβ/δ, were analyzed using qRT-PCR. The results indicated that PPARα expression was significantly upregulated in both *FSCN1*-knockdown and *FSCN1*-knockout cells, whereas PPARβ/δ expression showed no significant change (Fig. 4F). Western blot analysis further confirmed increased PPARα protein levels upon *FSCN1* suppression (Fig. 4G and Supplementary Fig. S6). Bioinformatics analysis revealed a negative correlation between FSCN1 and PPARα (encoded by *PPARA*) expression, and a positive correlation between PPARα (encoded by *PPARA*) and ACOX1 expression (Fig. 4B,C). Taken together, these findings demonstrate a consistent negative association between FSCN1 expression and the PPARα/ACOX1 axis in CRC cells. Given that PPARα is a well-characterized transcriptional activator of ACOX1, these coordinated molecular changes may contribute to impaired peroxisomal fatty acid oxidative catabolism and subsequent intracellular lipid accumulation in the context of dysregulated FSCN1 expression.

### 3.5 FSCN1 Expression Correlates with AKT/mTOR Pathway Activation in CRC Cells

**Figure 5:**
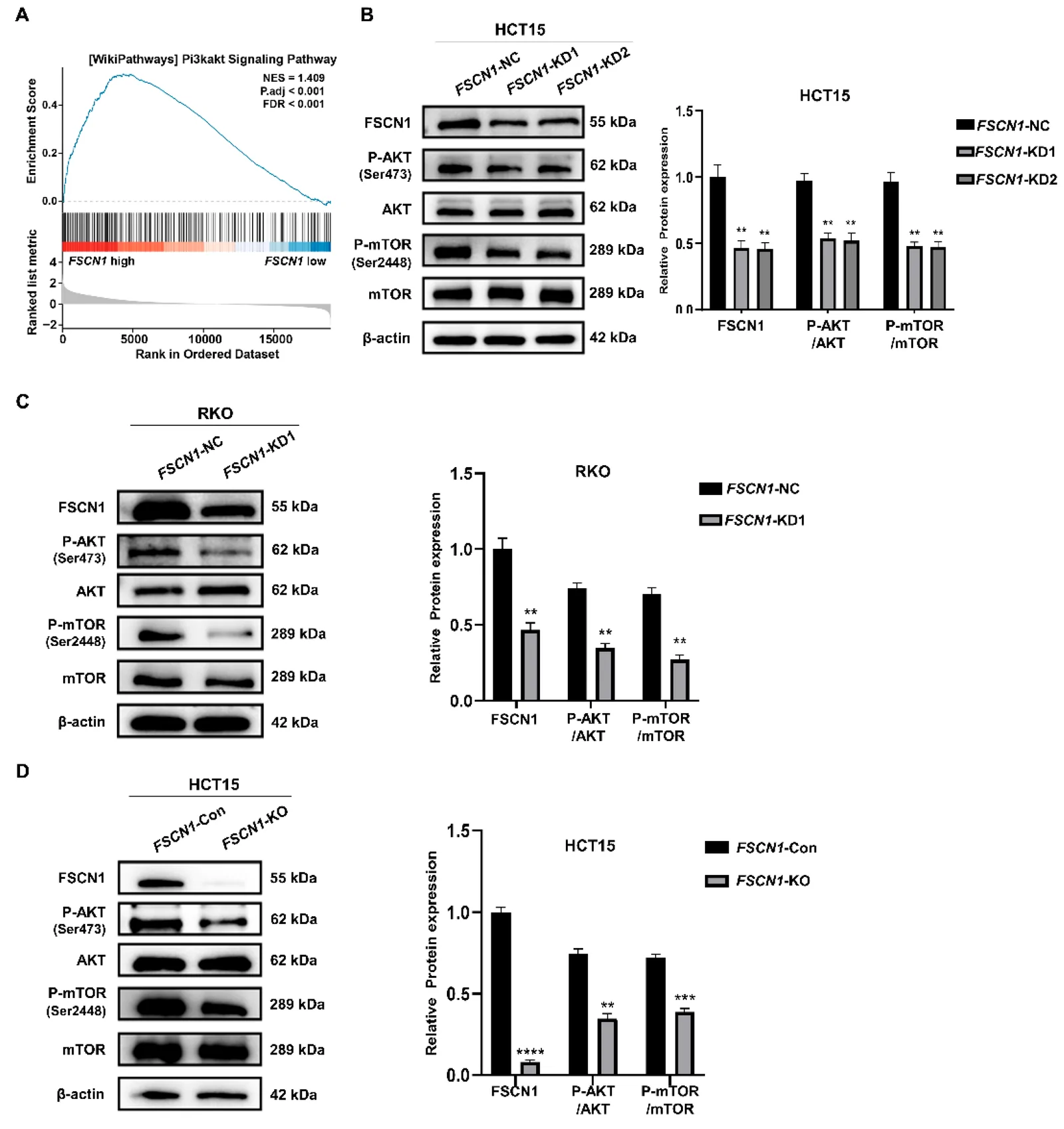
Association of FSCN1 expression with AKT/mTOR pathway activation. (**A**) GSEA (WikiPathways) of the TCGA-COADREAD cohort reveals significant activation of PI3K/AKT signaling in FSCN1-high tumors (FDR < 0.25, *p* < 0.05). (**B**–**D**) Western blot analysis of key AKT/mTOR pathway proteins in stable *FSCN1*-KD/KO cell lines and corresponding controls. Representative blot images from three independent experiments are shown on the left. The corresponding bar charts (on the right) quantify the average relative protein expression levels, calculated from the ImageJ densitometric analysis of the bands. β-actin served as the loading control. Data are presented as the mean ± standard deviation (SD) from three independent biological replicates (*n* = 3). **p < 0.01; ***p < 0.001; ****p < 0.0001. Abbreviations: GSEA, Gene Set Enrichment Analysis; KEGG, Kyoto Encyclopedia of Genes and Genomes; NES, normalized enrichment score; FDR, false discovery rate; FSСN1, Fascin actin-bundling protein 1; AKT, protein kinase B; p-AKT, phosphorylated protein kinase B; mTOR, mechanistic target of rapamycin; p-mTOR, phosphorylated mechanistic target of rapamycin; NC, negative control; KD, knockdown; KO, knockout; Con, control; β-actin, beta-actin.

The above results suggest that FSCN1-associated intracellular lipid accumulation may involve alterations both anabolic and catabolic branches of lipid metabolism. To explore potential molecular links underlying this association, GSEA was performed using TCGA-derived CRC tissue data stratified by FSCN1 expression levels. The GSEA results revealed a significant positive correlation between FSCN1 expression and enrichment of the AKT/mTOR signaling gene sets (Fig. 5A). Accumulating evidence has established that the AKT/mTOR signaling cascade serves a crucial regulator of energy metabolism [25] and is tightly coupled, to lipid homeostasis. These findings prompted us to hypothesize that the link between FSCN1 and lipid metabolism regulation may be related to AKT/mTOR signaling activity. To test this hypothesis, the protein levels of key pathway components was examined via Western blot analysis. In line with our hypothesis, *FSCN1* knockdown markedly attenuated the phosphorylation of AKT at Ser473 and mTOR at Ser2448 in CRC cells (Fig. 5B–D). Collectively, these data suggest that FSCN1 expression is positively associated with AKT/mTOR signaling activation in CRC cells.

### 3.6 FSCN1 Expression Correlates with p38 MAPK Pathway Activation in CRC Cells

**Figure 6:**
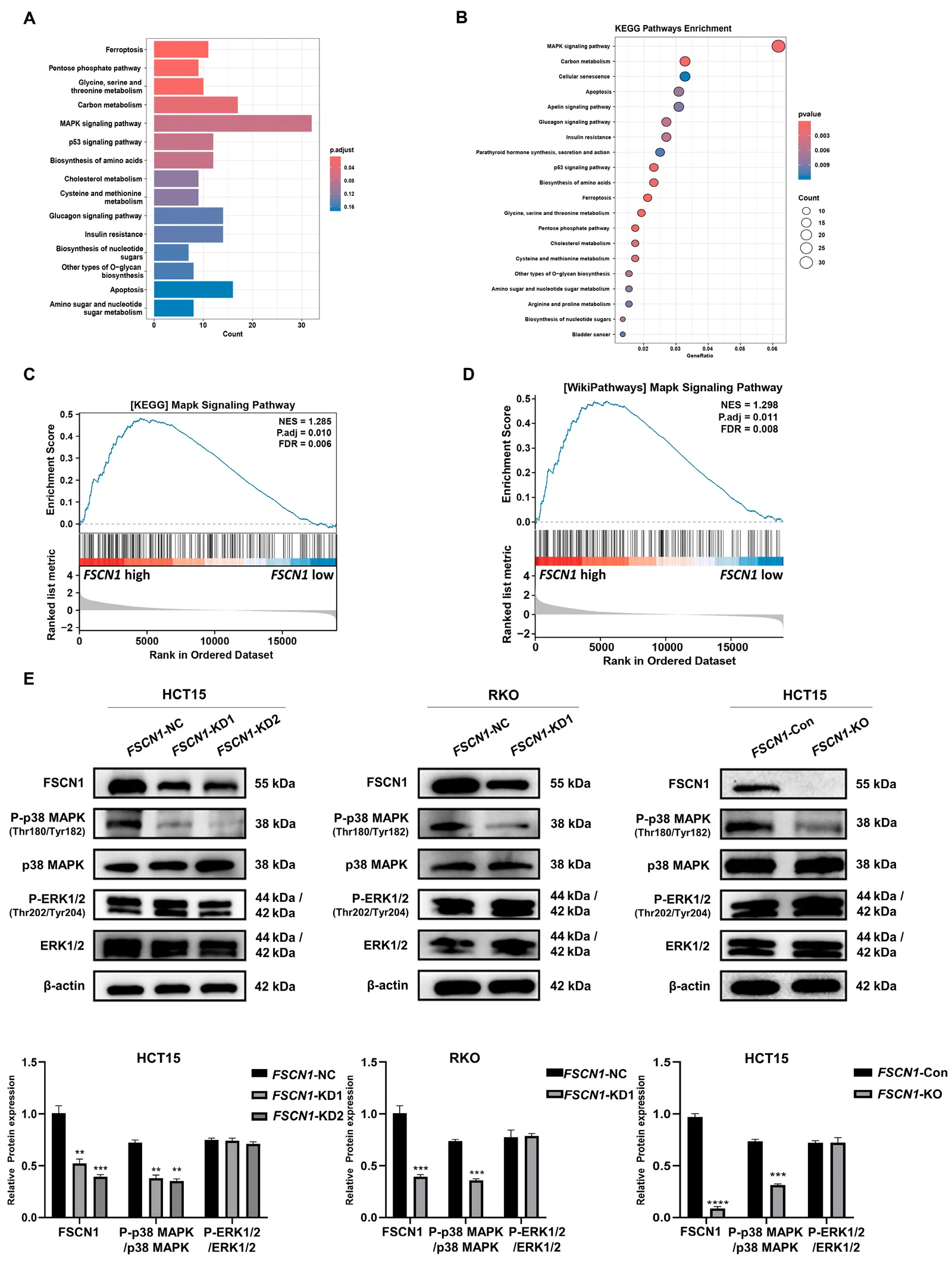
Association of FSCN1 expression with p38 MAPK pathway activation. (**A**,**B**) KEGG pathway analysis of differentially expressed genes (DEGs) from RNA-seq data of *FSCN1*-KD CRC cells identified MAPK signaling as the most significantly enriched pathway. (**C**,**D**) Concordant GSEA validation across experimental (RNA-seq: NES = 1.285, FDR = 0.006) and clinical (TCGA-COADREAD: NES = 1.298, FDR = 0.008) datasets confirmed FSCN1-related MAPK activation (FDR < 0.25 and *p* < 0.05 were considered statistically significant). (**E**) Western blot analysis revealed selective phosphorylation of p38 MAPK at Thr180/Tyr182 without altering ERK1/2 phosphorylation (Thr202/Tyr204) in stable FSCN1-KD/KO cell lines and corresponding controls, indicating isoform-specific pathway engagement. Representative blot images from three independent experiments are shown above. The corresponding bar charts (below) quantify the average relative protein expression levels, calculated from the ImageJ densitometric analysis of the bands. β-actin served as the loading control. Data are presented as the mean ± standard deviation (SD) from three independent biological replicates (*n* = 3). **p < 0.01; ***p < 0.001; ****p < 0.0001. Abbreviations: KEGG, Kyoto Encyclopedia of Genes and Genomes; GSEA, Gene Set Enrichment Analysis; NES, normalized enrichment score; FDR, false discovery rate; MAPK, mitogen-activated protein kinase; ERK, extracellular signal-regulated kinase; P-ERK1/2, phosphorylated extracellular signal-regulated kinase 1/2; P-p38 MAPK, phosphorylated p38 mitogen-activated protein kinase; NC, negative control; KD, knockdown; Con, control; *β*-actin, beta-actin.

To explore potential molecular links underlying FSCN1-associated alterations in cellular lipid metabolism, raw RNA-seq data from HCT15-*FSCN1*-KD cells [20] were reanalyzed, and KEGG pathway enrichment analysis was performed. The results revealed that DEGs in *FSCN1*-suppressed cells were most significantly enriched in the MAPK signaling pathway (Fig. 6A,B). Further GSEA of the same RNA-seq dataset stratified by FSCN1 expression levels confirmed that FSCN1 expression was associated with enrichment of MAPK pathway gene sets in CRC cells (Fig. 6C). Consistently, GSEA of TCGA-derived CRC tissue data also indicated a significant association between FSCN1 expression and MAPK pathway gene set enrichment (Fig. 6D). To validate these bioinformatics observations, key MAPK pathway components was examined via Western blot analysis. *FSCN1* inhibition in CRC cells significantly reduced phosphorylation of p38 MAPK at Thr180/Tyr182, whereas phosphorylation levels of extracellular signal-regulated kinase 1/2 (ERK1/2) at Thr202/Tyr204 showed no significant change (Fig. 6E). These results suggest that FSCN1 expression is positively associated with p38 MAPK signaling activation in CRC cells.

### 3.7 AKT/mTOR Signaling Correlates with FSCN1-Related Lipid Biosynthesis

**Figure 7:**
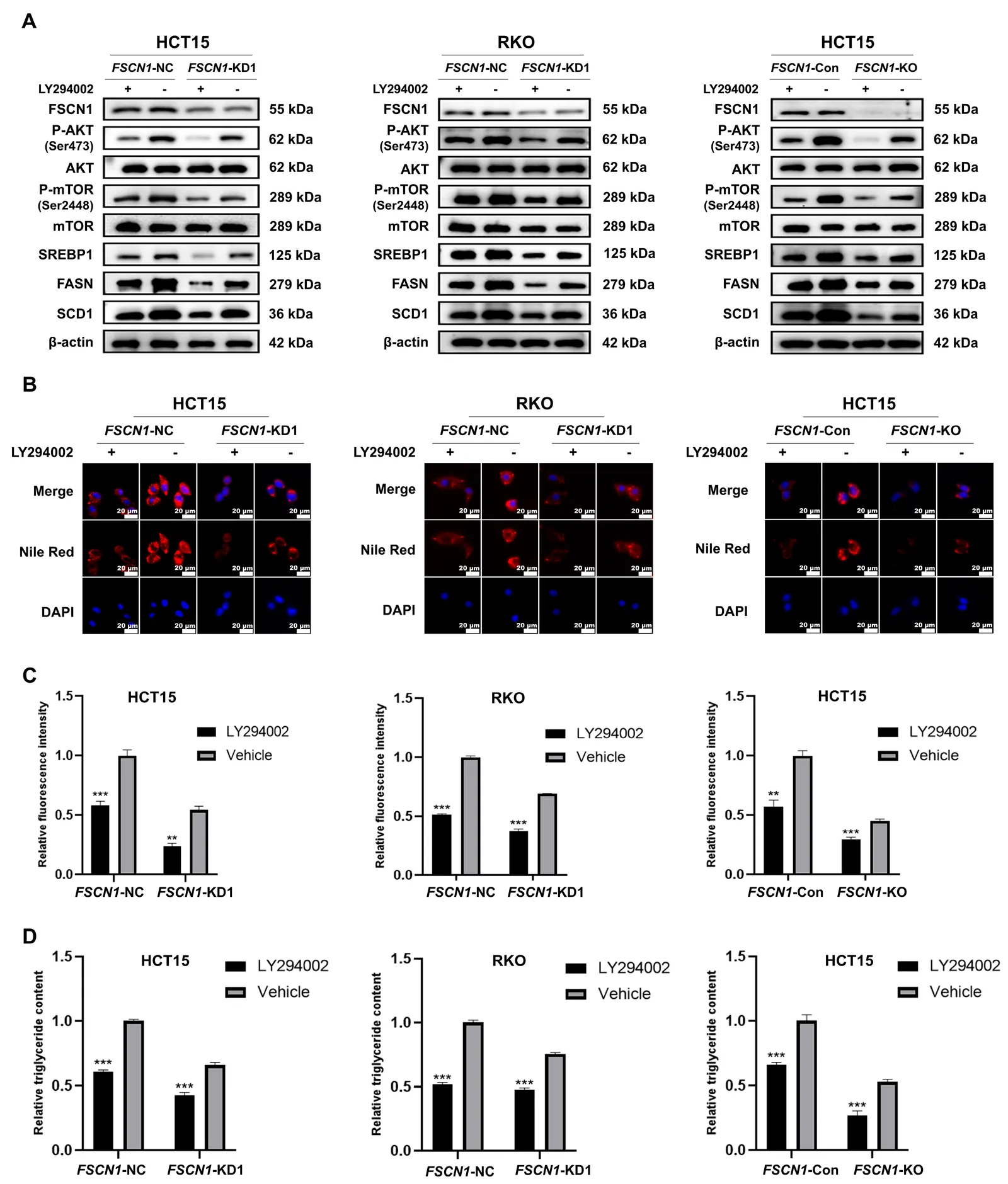
Association of AKT/mTOR signaling with FSCN1-related lipid biosynthesis. (**A**) Western blot analysis demonstrating dose-dependent suppression of AKT/mTOR signaling (phosphorylated AKT [p-AKT] and phosphorylated mTOR [p-mTOR]) and downstream lipogenic effectors (SREBP1, FASN, and SCD1) by LY294002 (20 μM) treatment. Corresponding quantitative densitometric analyses are shown in Supplementary Figs. S8–S10. (**B**) Representative fluorescence images of Nile Red (red fluorescence; a hydrophobic dye that specifically binds to intracellular lipid droplets and neutral lipids) and DAPI (blue fluorescence; a nuclear counterstain) double-staining in LY294002 (20 μM)-treated cells and corresponding vehicle-treated controls. Blinded quantitative analysis was performed for Nile Red staining. This staining reveals the overall distribution pattern of intracellular neutral lipids, and alterations in lipid droplet content were independently verified by biochemical triglyceride assays. Scale bar, 20 μm. (**C**) Relative Nile red fluorescence intensity of cells in (**B**), normalized to DAPI staining intensity. (**D**) Quantitative analysis of relative triglyceride content in LY294002-treated cells and corresponding vehicle-treated controls. β-actin served as the loading control. Data are shown as the mean ± standard deviation (SD) from three independent biological replicates (*n* = 3). **p < 0.01; ***p < 0.001. Abbreviations: NC, negative control; KD, knockdown; Con, control; KO, knockout; FSCN1, fascin actin-bundling protein 1; p-AKT, phosphorylated protein kinase B; AKT, protein kinase B; p-mTOR, phosphorylated mechanistic target of rapamycin; mTOR, mechanistic target of rapamycin; SREBP1, sterol regulatory element-binding protein 1; FASN, fatty acid synthase; SCD1, stearoyl-CoA desaturase 1; DAPI, 4′,6-diamidino-2-phenylindole.

Previous studies have shown that SREBP1 expression and activity are tightly controlled by multiple upstream signaling nodes [26,27], among which the AKT/mTOR cascade is a well-characterized regulator of lipid biosynthesis via the SREBP1/(FASN/SCD1) axis [28]. Based on preliminary findings, we hypothesized that the association between FSCN1 and SREBP1-driven lipid biosynthesis may involve the AKT/mTOR signaling pathway. To test this hypothesis, cells were treated with LY294002, a selective inhibitor of PI3K (phosphatidylinositol-3-kinase; the upstream kinase of this cascade) for 48 h, followed by qRT-PCR and Western blot analyses. qRT-PCR results showed that SREBP1 mRNA levels were significantly reduced following LY294002 treatment (Supplementary Fig. S7). Western blot analysis further revealed substantial decreases in phosphorylated AKT (p-AKT), phosphorylated mTOR (p-mTOR), SREBP1, FASN, and SCD1 protein levels in LY294002-treated cells compared with vehicle-treated controls (Fig. 7A and Supplementary Figs. S8–S10). Consistently, Nile Red staining and triglyceride quantification assays confirmed reduced lipid accumulation in the LY294002-treated cells relative to vehicle-treated controls (Fig. 7B–D). Collectively, these findings indicate that the AKT/mTOR signaling pathway is associated with FSCN1-linked upregulation of SREBP1 and downstream lipogenic enzymes, as well as intracellular lipid accumulation in CRC cells. Consistently, LY294002-mediated AKT/mTOR inhibition phenocopies the effects of FSCN1 loss on SREBP1, FASN, and SCD1 expression, lending preliminary support to a putative FSCN1→AKT/mTOR→SREBP1 regulatory relationship.

### 3.8 p38 MAPK Signaling Correlates with FSCN1-Related Oxidative Lipid Catabolism

**Figure 8:**
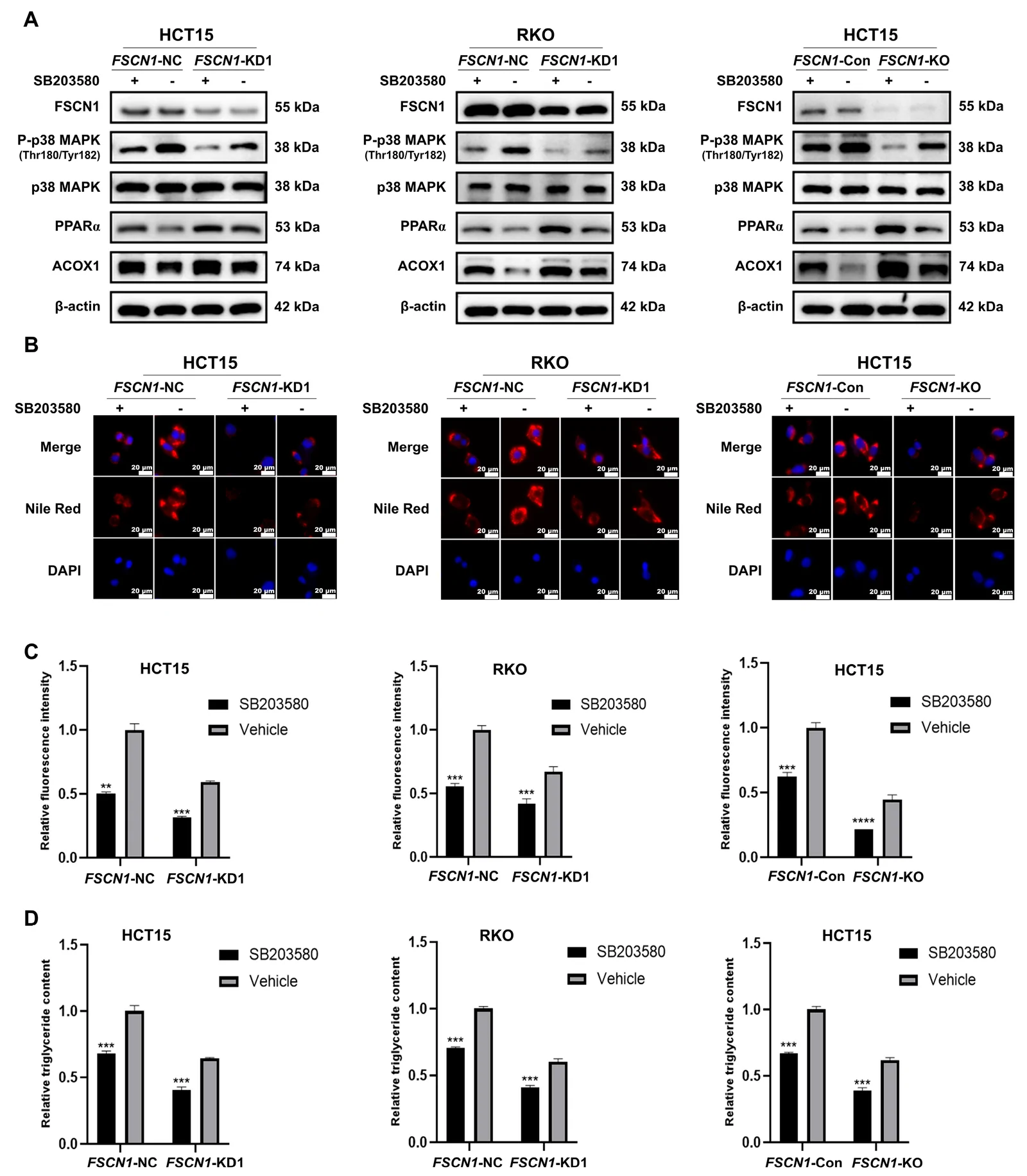
Association of p38 MAPK signaling with FSCN1-related peroxisomal FAO. (**A**) Western blot analysis showing dose-dependent suppression of the p38 MAPK signaling pathway (phosphorylated p38 MAPK [p-p38 MAPK]) and downstream lipolytic effectors (PPARα and ACOX1) by SB203580 (20 μM) treatment. Corresponding quantitative densitometric analyses are shown in Supplementary Figs. S12–S14. (**B**) Representative fluorescence images of Nile Red (red fluorescence; a hydrophobic dye that specifically binds to intracellular lipid droplets and neutral lipids) and DAPI (blue fluorescence; a nuclear counterstain) double-staining in SB203580 (20 μM)-treated cells and corresponding vehicle-treated controls. Blinded quantitative analysis was performed for Nile Red staining. Nile Red staining here illustrates the general trend of neutral lipid changes, with quantitative alterations in lipid droplets independently validated by triglyceride measurement. Scale bar, 20 μm. (**C**) Relative Nile red fluorescence intensity of cells in (**B**), normalized to DAPI staining intensity. (**D**) Quantitative analysis of relative triglyceride content in SB203580-treated cells and corresponding vehicle-treated controls. β-actin served as the loading control. Data are shown as the mean ± standard deviation (SD) from three independent biological replicates (*n* = 3). **p < 0.01; ***p < 0.001; ****p < 0.0001. Abbreviations: FSCN1, fascin actin-bundling protein 1; p-p38 MAPK, phosphorylated p38 mitogen-activated protein kinase; p38 MAPK, p38 mitogen-activated protein kinase; PPARα, peroxisome proliferator-activated receptor alpha; ACOX1, acyl-CoA oxidase 1; DAPI, 4′,6-diamidino-2-phenylindole; NC, negative control; KD, knockdown; Con, control; KO, knockout.

Previous studies have established that PPARα and PPARβ/δ regulate lipid metabolism by promoting FAO [29], and the p38 MAPK pathway has been reported to modulate cellular lipid oxidation through the PPARα/ACOX1 axis [12]. Based on preliminary findings, we hypothesized that the link between FSCN1 and PPARα-mediated lipid oxidation may involve the p38 MAPK signaling pathway. To validate this hypothesis, cells were treated with the p38 MAPK inhibitor SB203580 for 48 h, followed by parallel qRT-PCR and Western blot analyses. qRT-PCR results showed that PPARα mRNA levels were significantly increased following SB203580 treatment (Supplementary Fig. S11). Western blot analysis further demonstrated a marked reduction in phosphorylated p38 MAPK (p-p38 MAPK) protein levels, accompanied by elevated PPARα and ACOX1 protein expression in SB203580-treated cells compared with vehicle-treated controls (Fig. 8A and Supplementary Figs. S12–S14). In addition, Nile Red staining and triglyceride quantification assays showed decreased lipid accumulation in the SB203580-treated cells relative to vehicle-treated controls (Fig. 8B–D). Taken together, these data suggest that p38 MAPK signaling activity is associated with FSCN1-related downregulation of the PPARα/ACOX1 axis and the suppression of peroxisomal FAO, which may contribute to intracellular lipid accumulation in CRC cells. Consistently, SB203580-mediated p38 MAPK inhibition phenocopies the effects of FSCN1 depletion on PPARα/ACOX1 expression, lending preliminary support to a putative FSCN1→p38 MAPK⊣PPARα regulatory relationship.

### 3.9 Proposed Model of FSCN1-Related Lipid Metabolism Regulating in CRC Cells

Based on these findings, a working model is proposed in which FSCN1 expression is associated with coordinated alterations in lipid metabolism in CRC cells. Specifically, FSCN1 expression correlates with activation of the AKT/mTOR signaling pathway, upregulation of SREBP1, and elevated expression of its downstream lipogenic targets FASN and SCD1, concurrent with enhanced lipid biosynthesis. Concurrently, FSCN1 expression is associated with activation of the p38 MAPK pathway, downregulation of PPARα, and reduced expression of ACOX1, accompanied by suppressed lipid oxidation. Collectively, these coordinated changes—involving both enhanced lipogenesis and attenuated lipid catabolism—are linked to intracellular lipid accumulation and may contribute to malignant phenotypes and tumor progression in CRC.

**Figure 9:**
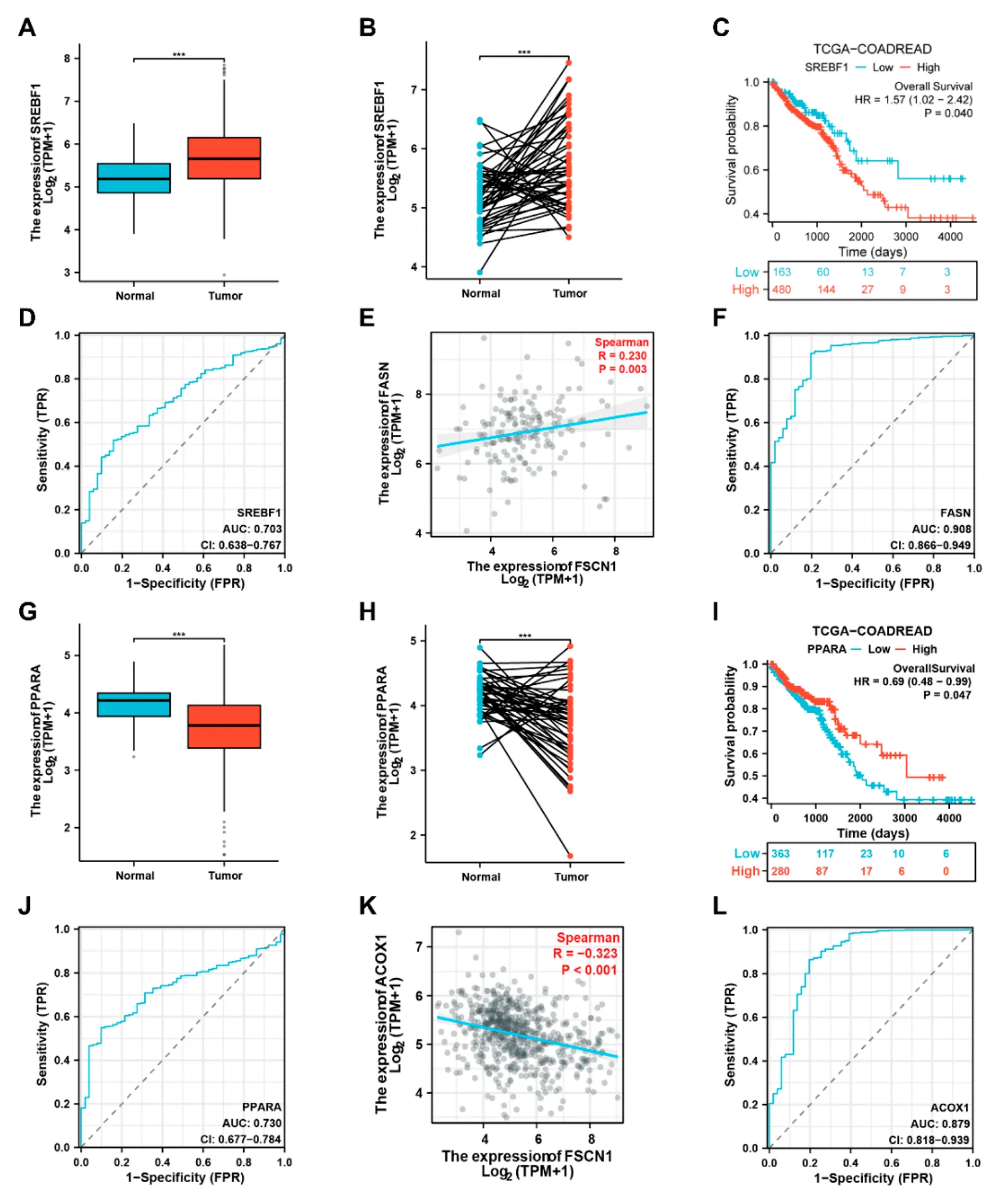
Potential diagnostic and prognostic value of the FSCN1-related axis in colorectal cancer (CRC). (**A**) Tumor-specific upregulation of SREBP1 (*SREBF1*) in TCGA CRC tissues compared with GTEx normal colorectal mucosal tissues (Normal = 51, Tumor = 644). (**B**) Tumor-specific upregulation of SREBP1 (*SREBF1*) in paired tumor/normal tissues from the same TCGA patients (n = 50 pairs). (**C**) Kaplan-Meier survival curves showing poorer overall survival in SREBP1 (*SREBF1*)-high patients (Low = 163, High = 480; patients grouped by median *SREBF1* expression; n = 643 total, 1 patient excluded due to missing follow-up data). (**D**) ROC curves demonstrating the diagnostic potential of SREBP1 (*SREBF1*) (AUC = 0.703, 95% CI 0.638–0.767) for distinguishing CRC from normal mucosa. (**E**) Scatter plot showing a positive correlation between FSCN1 and FASN mRNA expression levels using TCGA data. (**F**) Receiver operating characteristic (ROC) curves demonstrating the diagnostic potential of FASN (AUC = 0.908, 95% CI 0.866–0.949) for distinguishing CRC from normal mucosa. (**G**) Tumor-specific downregulation of PPARα (*PPARA*) in TCGA CRC tissues compared with GTEx normal colorectal mucosal tissues (Normal = 51, Tumor = 644). (**H**) Tumor-specific downregulation of PPARα (*PPARA*) in paired tumor/normal tissues from the same TCGA patients (n = 50 pairs). (**I**) Kaplan-Meier survival curves showing reduced overall survival in PPARα (*PPARA*)-low patients (Low = 363, High = 280; patients grouped by median *PPARA* expression; n = 643 total, 1 patient excluded due to missing follow-up data). (**J**) ROC curve demonstrating the diagnostic potential of PPARα (*PPARA*) (AUC = 0.730, 95% CI 0.677–0.784) for distinguishing CRC from normal mucosa. (**K**) Scatter plot showing a negative correlation between FSCN1 and ACOX1 mRNA expression levels using TCGA data. (**L**) ROC curve demonstrating the diagnostic potential of ACOX1 (AUC = 0.879, 95% CI 0.818–0.939) for distinguishing CRC from normal mucosa. ***p < 0.001. Abbreviations: FSCN1, fascin actin-bundling protein 1; SREBP1, sterol regulatory element-binding protein 1; FASN, fatty acid synthase; PPARα, peroxisome proliferator-activated receptor alpha; ACOX1, acyl-CoA oxidase 1; TCGA, The Cancer Genome Atlas; COADREAD, colon adenocarcinoma and rectal adenocarcinoma; TPM, transcripts per million; AUC, area under the curve; CI, confidence interval; HR, hazard ratio; TPR, true positive rate; FPR, false positive rate.

To further support this conclusion at the tumor tissue level, bioinformatics analyses were performed using public databases to evaluate the expression of key lipid metabolism regulators (SREBP1 and PPARα) and their potential as molecular markers for CRC diagnosis and prognosis. SREBP1 (*SREBF1*) expression was significantly higher in CRC tissues (*p* < 0.001; Fig. 9A), as confirmed in paired CRC/adjacent normal tissues (*p* < 0.001; Fig. 9B). Kaplan-Meier analysis showed that high SREBP1 (*SREBF1*) expression correlated with poor overall survival (*p* = 0.04; Fig. 9C). The AUC values for SREBP1 (*SREBF1*) and FASN were 0.703 (95% CI: 0.638–0.767) and 0.908 (95% CI: 0.866–0.949), respectively, indicating a strong diagnostic accuracy for differentiating CRC from normal tissues (Fig. 9D,F). Bioinformatics analysis further revealed a positive correlation between FSCN1 and FASN expression (Fig. 9E), consistent with the prior correlations between FSCN1/SREBP1 (*SREBF1*) (Fig. 3C) and SREBP1 (*SREBF1*)/FASN (Fig. 3D). Notably, PPARα (*PPARA*) and ACOX1 exhibited opposite expression trends (Fig. 9G–L), suggesting a potential association of FSCN1 expression with opposing alterations in lipid anabolic and catabolic pathways. These findings point to the potential clinical relevance of FSCN1 and its related pathway molecules as candidate diagnostic or prognostic markers for CRC.

**Figure 10:**
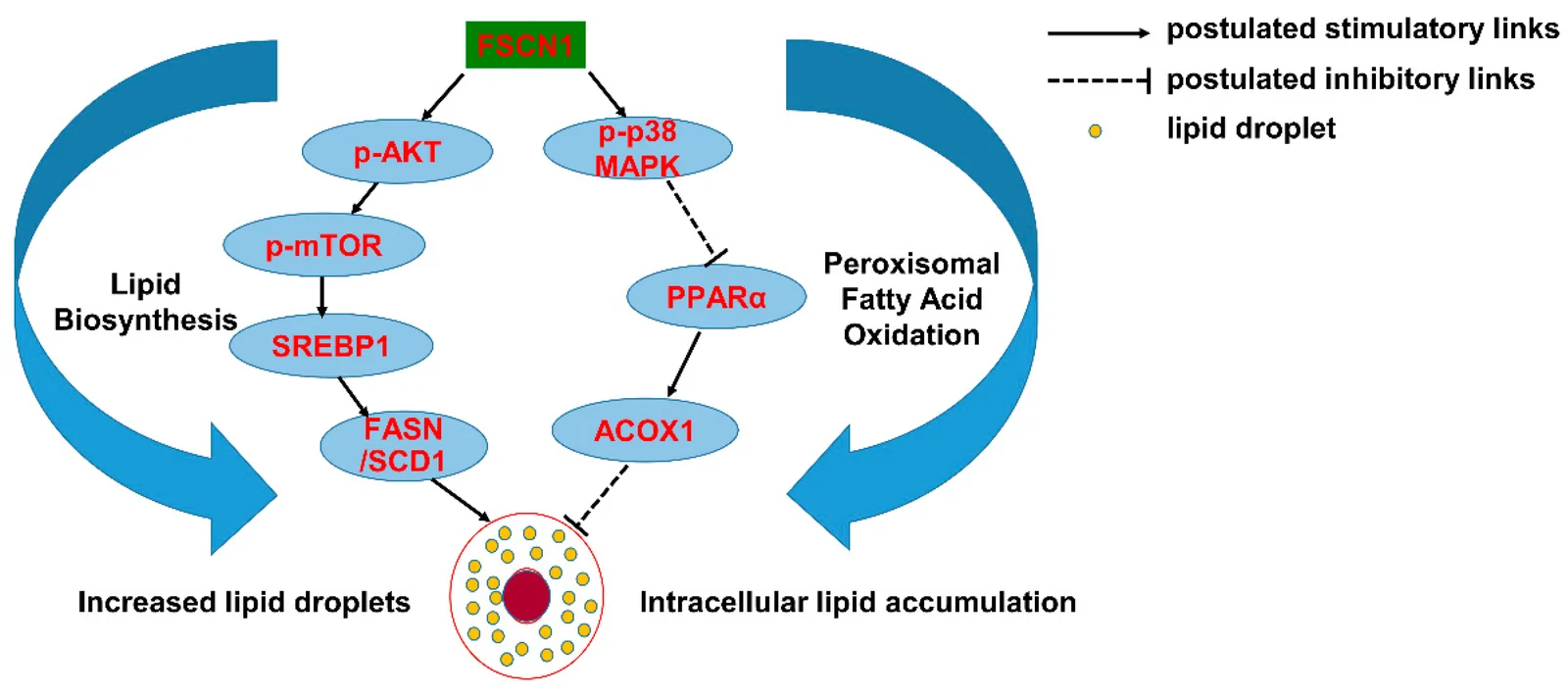
Proposed model of FSCN1-related lipid metabolic regulation in CRC. This proposed model illustrates associations between FSCN1 expression and lipid metabolic regulation in CRC cells. FSCN1 expression correlates with activation of the AKT/mTOR/SREBP1/(FASN/SCD1) axis (lipid biosynthesis) and suppression of the p38 MAPK/PPARα/ACOX1 pathway (peroxisomal fatty acid oxidation), and these coordinated molecular changes are associated with increased intracellular lipid droplet accumulation. Note: Solid arrows (→) indicate postulated stimulatory links; dashed lines with perpendicular bars (----⊣) indicate postulated inhibitory links; yellow dots represent lipid droplets. Abbreviations: FSCN1, fascin actin-bundling protein 1; p-AKT, phosphorylated protein kinase B; AKT, protein kinase B; p-mTOR, phosphorylated mechanistic target of rapamycin; mTOR, mechanistic target of rapamycin; SREBP1, sterol regulatory element-binding protein 1; FASN, fatty acid synthase; SCD1, stearoyl-CoA desaturase 1; p-p38 MAPK, phosphorylated p38 mitogen-activated protein kinase; p38 MAPK, p38 mitogen-activated protein kinase; PPARα, peroxisome proliferator-activated receptor alpha; ACOX1, acyl-CoA oxidase 1.

In summary, the coordinated changes involving the FSCN1/p38 MAPK/PPARα/ACOX1 and FSCN1/AKT/mTOR/SREBP1/(FASN/SCD1) pathways (schematically depicted in Fig. 10) suggest potential molecular correlates that may contribute to our understanding of CRC malignant progression.

## 4 Discussion

Cancer is a systemic metabolic disorder characterized by metabolic reprogramming during tumor initiation and progression [4,5,30,31]. As early as the 1920s, Otto Warburg discovered a form of metabolic reprogramming in tumor cells, aerobic glycolysis [32]. This study provides correlative evidence suggesting that FSCN1 expression is associated with lipid metabolism regulation and CRC progression, which may inform further functional investigations of FSCN1.

With respect to lipid anabolism, Zhang et al. have demonstrated that SREBP1 promotes the growth and metastasis of breast cancer both *in vitro* and *in vivo* [33]. PTPRO suppresses *de novo* lipogenesis by attenuating SREBP1 expression and its target lipogenic enzyme, acetyl-CoA carboxylase alpha (*ACC1*) [12]. In this study, we observed that FSCN1 expression is positively correlated with SREBP1, a transcription factor known to transcriptionally activate FASN and SCD1, and these coordinated changes are associated with tumor progression. A limitation of this study is that we do not provide direct evidence for the transcriptional or post-transcriptional regulation of SREBP1 by FSCN1. Based on the well-established AKT/mTOR/SREBP1 axis and prior work demonstrating that upstream regulators typically modulate SREBP1 via signaling cascades rather than direct physical interaction [12], we propose that FSCN1 regulates SREBP1 levels indirectly through this pathway. Future studies employing chromatin immunoprecipitation or protein turnover assays will be required to elucidate the precise underlying mechanism.

Regarding lipid catabolism, metabolic reprogramming in cancer involves not only mitochondrial β-oxidation but also peroxisomal fatty acid metabolism [34]. ACOX1 is the rate-limiting enzyme for peroxisomal fatty acid β-oxidation. Typically, very long-chain fatty acids (VLCFAs) are first oxidized by ACOX1 and downstream peroxisomal enzymes, and the resulting shorter-chain fatty acids are further catabolized in the mitochondria [35]. In colorectal adenocarcinoma (COAD) and other malignancies, peroxisomal lipid metabolism not only supports membrane biosynthesis and energy requirements but also mitigates oxidative stress, thereby facilitating cancer cell survival under metabolic stress [36]. Following peroxisomal β-oxidation, VLCFAs are typically shortened to long-chain fatty acids (LCFAs; ≤18 carbons). The transport of LCFAs into the mitochondria is regulated by CPT1A, a rate-limiting enzyme in FAO. In this study, inhibition of *FSCN1* in CRC cells was found to be associated with upregulated ACOX1 expression, which correlated with enhanced peroxisomal FAO and reduced tumor cell proliferation and metastasis. These findings are consistent with subsequent reports showing that ACOX1 downregulation in CRC is associated with intracellular lipid accumulation and may be linked to cancer cell growth and invasion [37]. However, no significant changes in CPT1A or CPT1B expression were observed during FSCN1-related metabolic alterations. This observation suggests that, although FAO involves multiple enzymatic steps, changes in ACOX1 expression—rather than CPT1A/B—may be more closely associated with the altered FAO rate in CRC cells under these conditions. This selective regulation is intriguing, as it suggests that FSCN1-driven p38 MAPK/PPARα signaling may preferentially modulate peroxisomal FAO (via ACOX1) rather than mitochondrial FAO (via CPT1A). Although the underlying mechanism remains unclear, potential explanations—such as differences in PPARα binding affinity to ACOX1 versus CPT1A promoters, or the involvement of specific co-regulators—warrant future investigation. Furthermore, future studies should investigate the potential crosstalk between peroxisomal and mitochondrial FAO in FSCN1-mediated metabolic regulation. Previous studies have demonstrated that PPARα regulates ACOX1 to promote tumor cell growth [12,38]. In line with this, our study in CRC cells showed that FSCN1 inhibition was associated with upregulated PPARα and ACOX1 expression, along with reduced malignant phenotypes. Notably, the PPARα/ACOX1 regulatory axis is not unique to FSCN1 signaling; other genes, such as PTPRO, also utilize this pathway to modulate lipid catabolism and suppress tumor progression [12]. While the roles of SREBP1 in regulating FASN/SCD1 and PPARα in regulating ACOX1 are well established [12,39], investigating their precise functions and interactions within the specific context of CRC lipid metabolism remains an important future direction.

It has been reported that p38 MAPK activation increases the risk of tumorigenesis and metastasis in various human cancers [40]. Previous studies have shown that FSCN1 can modulate MAPK pathway activity in different cancer types [41,42]. Building upon these findings, the present study further observed that FSCN1 expression is associated with p38 MAPK pathway activation, downregulation of PPARα, and decreased ACOX1 expression. These coordinated changes correlate with suppressed peroxisomal FAO, increased intracellular lipid accumulation, and enhanced malignant phenotypes in CRC, which is consistent with the aforementioned literature.

Multiple studies have shown that fatty acid metabolism participates in various pathological processes by regulating cancer cell proliferation, ferroptosis, immune evasion, metastasis, and drug resistance [31,43]. For example, microRNA-199a-3p activates the PI3K/AKT signaling pathway and upregulates MMP2 (matrix metallopeptidase 2) expression by regulating SCD1 expression, thereby inhibiting nasopharyngeal carcinoma metastasis [44]. In esophageal squamous cell carcinoma, the ARL5B-ROCK1-SREBP1 axis drives malignant progression by enhancing lipogenic reprogramming and promoting tumor cell proliferative and invasive phenotypes [45]. In this study, FSCN1 expression was observed to be associated with AKT/mTOR pathway activation, upregulation of SREBP1, and increased expression of FASN and SCD1. These coordinated changes correlate with enhanced lipid biosynthesis and malignant progression in CRC, consistent with previous reports [46,47]. Collectively, our findings demonstrate coordinated changes in lipid metabolic gene expression and cellular lipid accumulation that are consistent with metabolic reprogramming. However, future studies using stable-isotope tracer techniques will be required to directly measure *de novo* lipogenesis and fatty acid β-oxidation fluxes. Furthermore, the downstream molecular events accompanying these metabolic alterations remain to be elucidated and warrant further investigation.

Nevertheless, we acknowledge that this study has not clarified how FSCN1 activates AKT/mTOR and p38 MAPK, and its binding partners linking cytoskeletal remodeling to kinase activation remain unidentified. As an actin-bundling protein, FSCN1 regulates cytoskeletal dynamics and cellular mechanosensing. Specifically, the integrin-FAK axis is a likely mediator. FAK is required for AKT/mTOR activation in intestinal tumorigenesis [48], and integrin α5/FAK/AKT signaling promotes colorectal cancer progression [49]. FAK also triggers MAPK activation [50], and the Src-FAK cascade activates ERK1/2 and p38 MAPK in colorectal cancer cells [51]. These findings suggest that FSCN1 may act via integrin-FAK-mediated mechanosensing to stimulate downstream signaling, a hypothesis currently under active investigation in our laboratory.

Future rescue, gain-of-function, and actin bundling-deficient mutant studies are required to establish causality, confirm bidirectional regulation of lipid metabolic pathways, and determine bundling dependency of FSCN1-mediated phenotypes, while the contribution of FSCN1-driven lipid accumulation to CRC invasion/metastasis remains to be tested.

Beyond lipid metabolism, FSCN1 activates AKT and MAPK pathways [52], regulates glycolysis and mitochondrial oxidative phosphorylation [53], and modulates mitochondrial quality control via mitophagy [54], supporting its role as a master regulator of cancer cell metabolism. Additionally, we acknowledge that our Nile Red staining did not resolve discrete lipid droplets, and we will employ BODIPY 493/503 for precise morphological assessment in future studies. We also acknowledge that the observed increase in intracellular TG and LDs may arise from combined alterations in multiple lipid metabolic processes, and that dedicated flux-tracing studies will be required in future work to delineate the exact underlying mechanisms. Furthermore, a limitation of this study is the lack of multivariate Cox regression analysis for FSCN1 due to high IHC staining variability and prohibitive sample expansion costs; thus, large-scale prospective studies are needed to confirm its independent prognostic value in colorectal cancer.

In conclusion, this study demonstrates that FSCN1 drives CRC invasion and metastasis by modulating lipid metabolism. These results suggest that disrupting FSCN1-mediated metabolic dysregulation may offer a new therapeutic strategy for CRC and support the further evaluation of FSCN1 as a biomarker of aggressive disease.

## 5 Conclusions

In summary, this study reveals a novel mechanism in which FSCN1 functions as a key regulator of lipid metabolism in CRC, coordinating the AKT/mTOR/SREBP1/(FASN/SCD1) and p38 MAPK/PPARα/ACOX1 axes to fuel lipid accumulation and malignant progression. These findings validate FSCN1 as both a potential prognostic biomarker and a therapeutic target for invasive, metastatic CRC, and indicate that targeting FSCN1-governed lipid metabolic regulation represents a promising intervention strategy.

## Data Availability

The data that support the findings of this study are openly available in The Cancer Genome Atlas Program (TCGA) at https://portal.gdc.cancer.gov/ (dbGaP accession: phs000178; Supplementary Material S1: TCGA-COADREAD) and in the Genotype-Tissue Expression (GTEx) at https://gtexportal.org/home/ (dbGaP accession: phs000424). The TPM-normalized expression dataset generated from raw sequencing data is provided as Supplementary Material S2: TPM_normalize_COADREAD. All other original findings and materials presented in the study are included in the main text and supplementary figures with corresponding legends; further inquiries can be directed to the corresponding author.

## Supplementary Materials

The supplementary material is available online at https://www.techscience.com/doi/10.32604/or.2026.084987/s1.
