# Supplementary material for: FSCN1 Modulates Fatty Acid Metabolism and the Coordinated Activation of AKT/mTOR and p38 MAPK Pathways in Colorectal Cancer Cells
Source: Oncol Res. 2026 Sep 14;34(10):24. doi: 10.32604/or.2026.084987 (PMC13591365; doi:10.32604/or.2026.084987)
Supplement: Supplementary file 1 [file OncolRes-34-84987-s001.zip › TSP_OR_84987-s001.docx]

**Supporting Information**

**Supplementary Figure S1.** qRT-PCR analysis of FSCN1 mRNA levels normalized to β-actin in stable *FSCN1*-KD/KO cell lines and their corresponding negative controls. Data are presented as mean ± standard deviation (SD) from three independent biological replicates (*n* = 3). **P* < 0.05; ***P* < 0.01; ****P* < 0.001; *****P* < 0.0001.**Abbreviations**: NC, negative control; KD, knockdown; KO, knockout**.**

**Supplementary Figure S2.** Quantitative analysis of the Western blot results shown in Figure 2A. Relative FSCN1 protein levels (normalized to β‑actin) in stable *FSCN1*-KD/KO cell lines and their respective negative controls were determined by densitometric analysis (ImageJ) of the corresponding protein bands. Each bar represents the mean ± standard deviation (SD) from three independent biological replicates (*n* = 3). **P* < 0.05; ***P* < 0.01; ****P* < 0.001; *****P* < 0.0001. **Abbreviations**: Con, control; KD, knockdown; KO, knockout; NC, negative control.

**Supplementary Figure S3.** Quantitative analysis of the Western blot results shown in Figure 2C. Relative protein levels of FSCN1, FASN, and SCD1 (normalized to β-actin) in stable *FSCN1*-KD/KO cell lines and their respective negative controls were determined by densitometric analysis (ImageJ) of the corresponding protein bands. Each bar represents the mean ± standard deviation (SD) from three independent biological replicates (*n* = 3). **P* < 0.05; ***P* < 0.01; ****P* < 0.001; *****P* < 0.0001. **Abbreviations**: Con, control; KD, knockdown; KO, knockout; NC, negative control.

**Supplementary Figure S4.** Quantitative analysis of the Western blot results shown in Figure 3B. Relative protein levels of FSCN1 and SREBP1 (normalized to β-actin) in stable *FSCN1*-KD/KO cell lines and their respective negative controls were determined by densitometric analysis (ImageJ) of the corresponding protein bands. Each bar represents the mean ± standard deviation (SD) from three independent biological replicates (*n* = 3). **P* < 0.05; ***P* < 0.01; ****P* < 0.001; *****P* < 0.0001. **Abbreviations**: Con, control; KD, knockdown; KO, knockout; NC, negative control.

**Supplementary Figure S5.** Quantitative analysis of the Western blot results shown in Figure 4E. Relative protein levels of FSCN1, ACOX1, and CPT1A (normalized to β-actin) in stable *FSCN1*-KD/KO cell lines and their respective negative controls were determined by densitometric analysis (ImageJ) of the corresponding protein bands. Each bar represents the mean ± standard deviation (SD) from three independent biological replicates (*n* = 3). **P* < 0.05; ***P* < 0.01; ****P* < 0.001; *****P* < 0.0001.**Abbreviations**: Con, control; KD, knockdown; KO, knockout; NC, negative control.

**Supplementary Figure S6.** Quantitative analysis of the Western blot results shown in Figure 4G. Relative protein levels of FSCN1 and PPARα (normalized to β-actin) in stable *FSCN1*-KD/KO cell lines and their respective negative controls were determined by densitometric analysis (ImageJ) of the corresponding protein bands. Each bar represents the mean ± standard deviation (SD) from three independent biological replicates (*n* = 3). **P* < 0.05; ***P* < 0.01; ****P* < 0.001; *****P* < 0.0001.**Abbreviations**: Con, control; KD, knockdown; KO, knockout; NC, negative control.

**Supplementary Figure S7.** qRT-PCR analysis of SREBP1 (*SREBF1*) mRNA levels normalized to β-actin in stable *FSCN1*-KD/KO cell lines and their corresponding negative controls following treatment with LY294002 or vehicle control. Data are presented as mean ± standard deviation (SD) from three independent biological replicates (*n* = 3). **P* < 0.05; ***P* < 0.01; ****P* < 0.001; *****P* < 0.0001.**Abbreviations**: NC, negative control; KD, knockdown; KO, knockout.

**Supplementary Figure S8.** Quantitative analysis of the Western blot results shown in the first column of Figure 7A. Relative protein levels of FSCN1, p-AKT/AKT, p-mTOR/mTOR, SREBP1, FASN, and SCD1 (normalized to β-actin) in stable HCT15-*FSCN1*-KD cell lines and their corresponding negative controls following treatment with LY294002 or vehicle control were determined by densitometric analysis (ImageJ) of the corresponding protein bands. Each bar represents the mean ± standard deviation (SD) from three independent biological replicates (*n* = 3). **P* < 0.05; ***P* < 0.01; ****P* < 0.001; *****P* < 0.0001.**Abbreviations**: KD, knockdown; NC, negative control.

**Supplementary Figure S9.** Quantitative analysis of the Western blot results shown in the second column of Figure 7A. Relative protein levels of FSCN1, p-AKT/AKT, p-mTOR/mTOR, SREBP1, FASN, and SCD1 (normalized to β-actin) in stable RKO-*FSCN1*-KD cell lines and their corresponding negative controls following treatment with LY294002 or vehicle control were determined by densitometric analysis (ImageJ) of the corresponding protein bands. Each bar represents the mean ± standard deviation (SD) from three independent biological replicates (*n* = 3). **P* < 0.05; ***P* < 0.01; ****P* < 0.001; *****P* < 0.0001.**Abbreviations**: KD, knockdown; NC, negative control.

**Supplementary Figure S10.** Quantitative analysis of the Western blot results shown in the third column of Figure 7A. Relative protein levels of FSCN1, p-AKT/AKT, p-mTOR/mTOR, SREBP1, FASN, and SCD1 (normalized to β-actin) in stable HCT15-*FSCN1*-KO cell lines and their corresponding negative controls following treatment with LY294002 or vehicle control were determined by densitometric analysis (ImageJ) of the corresponding protein bands. Each bar represents the mean ± standard deviation (SD) from three independent biological replicates (*n* = 3). **P* < 0.05; ***P* < 0.01; ****P* < 0.001; *****P* < 0.0001. **Abbreviations**: Con, control; KO, knockout.

**Supplementary Figure S11.** qRT-PCR analysis of PPARα mRNA levels normalized to β-actin in stable *FSCN1*-KD/KO cell lines and their corresponding negative controls following treatment with SB203580 or vehicle control. Data are presented as mean ± standard deviation (SD) from three independent biological replicates (*n* = 3). **P* < 0.05; ***P* < 0.01; ****P* < 0.001; *****P* < 0.0001.**Abbreviations**: NC, negative control; KD, knockdown; KO, knockout.

**Supplementary Figure S12.** Quantitative analysis of the Western blot results shown in the first column of Figure 8A. Relative protein levels of FSCN1, p-p38 MAPK/p38 MAPK, PPARα, and ACOX1 (normalized to β-actin) in stable HCT15-*FSCN1*-KD cell lines and their corresponding negative controls following treatment with SB203580 or vehicle control were determined by densitometric analysis (ImageJ) of the corresponding protein bands. Each bar represents the mean ± standard deviation (SD) from three independent biological replicates (*n* = 3). **P* < 0.05; ***P* < 0.01; ****P* < 0.001; *****P* < 0.0001.**Abbreviations**: KD, knockdown; NC, negative control.

**Supplementary Figure S13.** Quantitative analysis of the Western blot results shown in the second column of Figure 8A. Relative protein levels of FSCN1, p-p38 MAPK/p38 MAPK, PPARα, and ACOX1 (normalized to β-actin) in stable RKO-*FSCN1*-KD cell lines and their corresponding negative controls following treatment with SB203580 or vehicle control were determined by densitometric analysis (ImageJ) of the corresponding protein bands. Each bar represents the mean ± standard deviation (SD) from three independent biological replicates (*n* = 3). **P* < 0.05; ***P* < 0.01; ****P* < 0.001; *****P* < 0.0001. **Abbreviations**: KD, knockdown; NC, negative control.

**Supplementary Figure S14.** Quantitative analysis of the Western blot results shown in the third column of Figure 8A. Relative protein levels of FSCN1, p-p38 MAPK/p38 MAPK, PPARα, and ACOX1 (normalized to β-actin) in stable HCT15-*FSCN1*-KO cell lines and their corresponding negative controls following treatment with SB203580 or vehicle control were determined by densitometric analysis (ImageJ) of the corresponding protein bands. Each bar represents the mean ± standard deviation (SD) from three independent biological replicates (*n* = 3). **P* < 0.05; ***P* < 0.01; ****P* < 0.001; *****P* < 0.0001. **Abbreviations**: Con, control; KO, knockout.
